# NF‐Y‐dependent regulation of glutamate receptor 4 expression and cell survival in cells of the oligodendrocyte lineage

**DOI:** 10.1002/glia.23446

**Published:** 2018-04-27

**Authors:** Ghazala Begum, Masahiro Otsu, Usman Ahmed, Zubair Ahmed, Adam Stevens, Daniel Fulton

**Affiliations:** ^1^ Neuroscience and Ophthalmology Research Group Institute of Inflammation and Ageing, College of Medical and Dental Sciences, University of Birmingham Edgbaston Birmingham B15 2TT United Kingdom; ^2^ Division of Developmental Biology & Medicine, Faculty of Biology, Medicine & Health University of Manchester, Manchester Academic Health Sciences Centre Manchester M13 9PL United Kingdom

**Keywords:** AMPA receptor, CCAAT, excitotoxicity, GluA4, Gria4, NF‐Yb, oligodendrocyte precursor cell, Oli‐neu, transcriptional regulation

## Abstract

Glutamate receptor subunit 4 (GluA4) is highly expressed by neural cells sensitive to excitotoxicity, and is the predominant subunit expressed by oligodendrocyte precursor cells (OPC) during a key period of vulnerability to hypoxic‐ischemic injury. Therefore, transcriptional networks downstream of excitotoxic GluA4 activation represent a promising area for therapeutic intervention. In this work, we identify the CCAAT binding transcription factor NF‐Yb as a novel transcriptional regulator of *Gria4* (GluA4 gene), and a controller of excitotoxic death in the oligodendroglial lineage. We describe a novel regulatory region within *Gria4* containing CCAAT sequences whose binding by NF‐Yb is regulated by excitotoxicity. Excitotoxicity‐induced alterations in NF‐Yb binding are associated with changes in *Gria4* transcription, while knockdown of NF‐Yb alters the transcription of reporter constructs containing this regulatory region. Data from immortalized and primary OPC reveal that RNAi and pharmacological disruption of NF‐Yb alter *Gria4* transcription, with the latter inducing apoptosis and influencing a set of apoptotic genes similarly regulated during excitotoxicity. These data provide the first definition of a *trans‐acting* mechanism regulating *Gria4*, and identify the NF‐Y network as a potential source of pharmacological targets for promoting OPC survival.

## 1 INTRODUCTION

Oligodendrocyte precursor cells (OPC) express AMPA‐type glutamate receptors (AMPAR) whose activation in the healthy CNS has been shown to influence various functions including migration, proliferation, differentiation and survival (Fannon, Tarmier, & Fulton, 2015; Gallo et al., 1996; Gudz, Komuro, & Macklin, 2006; Kougioumtzidou et al., 2017; Yuan, Eisen, McBain, & Gallo, 1998). AMPAR also mediate injury to these cells under pathological conditions. In the developing rodent CNS, hypoxic‐ischemic excitotoxicity disrupts the formation of myelin through excitotoxic actions on OPC AMPAR (Follett, Rosenberg, Volpe, & Jensen, 2000), while in the adult CNS, inflammatory white matter injury and associated neurological dysfunctions, are linked to AMPAR‐mediated injury responses (Groom, Smith, & Turski, 2003; Kanwar, Kanwar, & Krissansen, 2004; Pitt, Werner, & Raine, 2000).

AMPAR are heterotetrameric assemblies containing various arrangements of GluA subunits 1–4 (GluA1–4). OPC express all four subunits, although GluA1 represents a minor contributor compared with GluA2–4 *in vitro* (Hossain, Liu, Fragoso, & Almazan, 2014; Itoh et al., 2002), and appears to be entirely absent from OPC *in vivo* (Kougioumtzidou et al., 2017). Activation of OPC AMPAR provokes an influx of Ca^2+^ (Ge et al., 2006; Haberlandt et al., 2011; Hamilton, Vayro, Wigley, & Butt, 2010; Itoh et al., 2002) that can mediate excitotoxic injury *in vitro* (Alberdi, Sanchez‐Gomez, Marino, & Matute, 2002; Deng, Rosenberg, Volpe, & Jensen, 2003; Li & Stys, 2000; Sanchez‐Gomez & Matute, 1999). These observations suggest that a substantial number of OPC AMPAR lack GluA2 subunits since inclusion of this subunit limits the permeability of AMPAR to Ca^2+^ (Geiger et al., 1995; Hollmann, Hartley, & Heinemann, 1991). In support of this, cultured OPC express high levels of GluA3 and 4 (Hossain et al., 2014; Itoh et al., 2002) which may assemble to form Ca^2+^ permeable AMPAR, and GluA4 is the predominant subunit expressed by OPC in the developing white matter of rodents and humans (Talos, Fishman, et al., 2006; Talos, Follett, et al., 2006). Importantly, the timing of GluA4 expression in these systems corresponds with an established window of vulnerability during which OPC are selectively injured by hypoxic‐ischemic conditions (Back et al., 2002; Back et al., 2001; reviewed in Fern, Matute, & Stys, 2014), and GluA4 is highly expressed in neural cells vulnerable to excitotoxic cell death (Page & Everitt, 1995). GluA4 signalling is therefore strongly connected to excitotoxicity.

Excitotoxic injury induces OPC and oligodendrocyte cell death through stress‐induced apoptotic pathways involving the Bcl‐2 family (Ness, Romanko, Rothstein, Wood, & Levison, 2001; Ness, Scaduto, & Wood, 2004; Sanchez‐Gomez, Alberdi, Ibarretxe, Torre, & Matute, 2003; Sanchez‐Gomez, Alberdi, Perez‐Navarro, Alberch, & Matute, 2011; Simonishvili, Jain, Li, Levison, & Wood, 2013). These processes are tightly regulated by the expression of pro‐ and anti‐apoptotic Bcl‐2 genes (Kumar & Cakouros, 2004; Riley, Sontag, Chen, & Levine, 2008), thus the transcriptional networks stimulated by excitotoxic injury represent promising targets for therapies aiming to reduce excitotoxic injury and cell death. In the context of OPC the transcriptional events associated with GluA4 are of particular interest due to its prominent expression in these cells, and its links to the induction of excitotoxic cell death (Page & Everitt, 1995; Santos et al., 2006). Based on this premise we used an excitotoxic injury model in the Oli‐neu cell line (Jung et al., 1995) and primary OPC (pOPC) to identify subunit B of the nuclear factor Y complex (NF‐Yb) as a regulator of GluA4 transcription and cell survival in oligodendroglia. Using a combination of ChiP, qPCR, Western blot and reporter assays we show that excitotoxic AMPAR stimulation alters NF‐Yb binding to a novel *Gria4* regulatory region, leading to complementary alterations in the levels of GluA4 mRNA and protein. We also provide data highlighting the therapeutic potential of the NF‐Y transcriptome, with siRNA and pharmacological‐mediated disruption of the NFY pathway compromising oligodendroglial viability and regulating similar apoptotic genes to those influenced by excitotoxic injury.

## 2 MATERIALS AND METHODS

### 2.1 Cell culture

Oli‐neu cells were kindly provided by Prof Jacqueline Trotter (University of Mainz). Oli‐neu cells were cultured in Sato medium containing 1% horse serum (Trotter, Bitter‐Suermann, & Schachner, 1989) and grown in 5% CO_2_ at 37°C. All experiments were carried out with cells at passage 5 after thawing. Cultures of pOPC were prepared from the neocortices of C57BL6/J mice aged 1–4 days using the protocol described by O'Meara, Ryan, Colognato, and Kothary (2011). Mixed glial cultures were maintained for 9 days (5% CO_2_ at 37°C) in DMEM containing 10% FBS and insulin (2 µg/ml) before isolation of pOPC by the shake‐off method. Isolated pOPC were seeded into poly‐l‐Lysine coated culture wells at a density of 1.0–5.0 × 10^5^ cells/well and maintained in OPC medium. OPC medium consisted of DMEM:F12 supplemented with 1% N2, 1% B27, 2 mM l‐Glutamine, 1% PC‐ST, 20 ng/ml rhFGF2, 100 ng/ml IGF1, 20 ng/ml PDGF‐AA, and 3.6 ng/ml Hydrocortisone. pOPC cultures were used for experiments between 1 and 2 days *in vitro*.

### 2.2 Drugs

Stock solutions of L‐glutamate (100 mM, Sigma‐Aldrich Company Ltd, Dorset, UK) and AMPA (100 mM, Abcam, Cambridge, UK) were prepared in distilled water, while cyclothiazide (CTZ, 100 mM, Bio‐Techne Ltd/R&D Systems, Abingdon, UK) and Garcinol (10 mM, Tocris Bioscience, Abingdon, UK) were prepared in DMSO. Drugs were diluted in Sato medium for application to Oli‐neu cells or OPC medium for experiments using pOPC. AMPA was applied with CTZ (AMPA/CTZ; both at 100 µM) for 5 hr to induce excitotoxicity (Alberdi et al., 2002), or at 200 µM (without CTZ) for 24 hr for non‐pathological AMPAR stimulation (AMPA_24h_; Yuan et al., 1998). In experiments using pOPC CTZ was applied at 50 µM. Garcinol was used at 10 µM for gene expression, Western blot, and ChIP experiments. This concentration was selected based on pilot studies examining treatment with log concentrations of Garcinol (1–1,000 µM), in which concentrations >10 µM caused catastrophic cell detachment and death (data not shown). BrdU (Sigma‐Aldrich Company Ltd) stocks were prepared as described by Fannon et al. (2015).

### 2.3 Trypan blue cell viability assay

For viability assays Oli‐neu were seeded into 6‐well plates at a density of 5 × 10^5^ cells/well, while pOPC were cultured in 12‐well plates at a density of 5 × 10^4^ cells/well. 24 hr after seeding cells were treated with l‐glutamate, CTZ, or AMPA/CTZ for 5 hr, then trypsinized, stained with 0.04% trypan blue (Sigma‐Aldrich) and counted on a haemocytometer to quantify % viability. Data were collected in triplicate from five independent cell cultures by an experimenter masked to the treatment.

### 2.4 Antibodies

Antibodies used in this study are detailed in Supporting Information Table S1. Specificity for immunopositive signals was determined in control samples incubated with secondary antibodies alone.

### 2.5 Immunocytochemistry, plasma membrane labelling and imaging

Immunofluorescent staining was performed using a protocol previously used in our lab to label brain slices (Fannon et al., 2015). The protocol was adapted for use on Oli‐neu cells as follows. Oli‐neu were grown in 8‐well chamber slides (Corning Falcon, Wiesbaden, Germany) at a density of 3 × 10^4^ cells/well, and the fixation, blocking, and secondary antibody incubation steps were reduced to 20 min, 1 hr, and 1 hr, respectively. Chamber slides were coverslipped with Vectashield containing DAPI (Vector Laboratories, Peterborough, UK). CellMask Deep Red (Molecular Probes, Eugene, OR; Thermo Fisher Scientific, Waltham, MA) was used to localize GluA4 to plasma membranes of Oli‐neu cells. Live cells were incubated in CellMask (2.5 µl/ml) diluted in Sato medium for 5 min (5% CO_2,_ 37°C) and immediately fixed in PFA, permeabalized with an optimized protocol (0.2% Tween‐20 for 10 min) which preserved CellMask signal while allowing reasonable labeling of GluA4. GluA4 was then detected as described above, except for omission of Triton‐X100 in the blocking and carrier solutions. Slides were imaged on an inverted microscope (Zeiss Axiovert 200, Zeiss, Oberkochen, Germany) equipped with a differential spinning disk confocal module as described previously (Fannon et al., 2015).Objectives used were 20× (Air, 0.5 N.A.) and 100× (oil, 1.3 N.A.). AMPAR immunofluorescence was imaged in confocal mode, while proliferation studies were imaged in the wide‐field configuration. Cleaved caspase‐3 labeling was imaged at 20× on Axioplan 200 epi‐fluorescent microscope equipped with an AxioCam HRc (all from Zeiss, Herefordshire, UK).

### 2.6 Proliferation and apoptosis studies

Cells were seeded into 8‐well chamber slides at a density of 3 × 10^5^ and allowed to settle for 24 hr prior to treatment with AMPA/CTZ, followed by exposure to BrdU (10 µM) for 7 hr. For AMPA_24h_ experiments BrdU treatments were introduced during the last 7 hr of treatment. BrdU was detected as previously described (Fannon et al., 2015) and proliferation quantified as the % DAPI^+^/BrdU^+^ cells from five randomly selected fields/well from seven to eight independent well replicates. Apoptosis was examined by immunofluorescent labelling for cleaved Caspase3 24 hr after treatment of cells with Garcinol (5 hr, 10 µM), or the vehicle control (DMSO, 0.001%). Data were quantified as % cleaved caspase‐3^+^/DAPI^+^ nuclei from four randomly selected areas of each well (*n* = 4/group).

### 2.7 GcCamp5 Ca^2+^ imaging

A recombinant Semlikiforest Virus (SFV) vector derived from strain A7(74) was constructed to deliver GcAMP5 (Akerboom et al., 2012) to Oli‐neu cells (Ehrengruber et al., 2003; Fannon et al., 2015). GcAMP5 cDNA (gift from Dr Lin Tian, University of California, Davis) was cloned into pSFV(A774nsP) and infectious SFV(A774nsP)‐GcAMP5 (SFV‐GcAMP5) particles generated in BHK cells using method a described by Ehrengruber, Schlesinger, and Lundstrom (2011). Oli‐neu cells in 35 mm imaging dishes (MatTek Corporation, Ashland, MA) were infected by treatment with Sato medium containing a 1:100 dilution of the un‐purified SFV‐GcAMP5 preparation (7.86 × 10^5^ infectious units/ml, 1 hr, 37°C/5% CO2). Cells were imaged the following day using the spinning disk module in confocal mode (20×, 485 nm excitation filter, 560 nm emission filter). Cells were perfused with medium *via* a peristaltic pump enabling delivery and removal of CTZ and AMPA/CTZ solutions. Solutions within the imaging dish were maintained at 34°C via an inline heater (Bioscience Tools, San Diego, CA) coupled to the perfusion setup. GcAMP5 signals were captured one frame/10 s through the following sequence: 5 min CTZ alone (baseline), 5 min AMPA/CTZ, 5 min CTZ alone. Mean signal intensity was measured using ImageJ (Schneider, Rasband, & Eliceiri, 2012), and changes in GcAMP5 quantified by normalizing to the mean baseline fluorescence (ΔF/F).

### 2.8 RNA and protein extraction

Five independent cultures of Oli‐neu cells were seeded into T25 (1 × 10^6^ cells/flask) in duplicate and left to adhere overnight. Duplicate flasks received the following treatment pairings: CTZ alone or AMPA/CTZ; Control Sato or AMPA_24h_; DMSO control or Garcinol (see Section 2.2 for treatment durations). Trypsinized samples were divided, washed with ice cold PBS, and RNA extracted from one sample using the RNeasy mini kit (Qiagen, Manchester, UK).The other sample was incubated for 30 min on ice with protein lysis buffer consisting of 20 nM Tris HCL pH 7.4, 150 mM NaCl, 1 mM EDTA, 0.5 mM EGTA, 1% Igepal and 5 µl/ml of protease inhibitor. Protein levels were then quantified using the RC/DC assay (Bio‐Rad, Hemel Hempstead, UK) and RNA/protein samples were stored at −70°C pending further analysis. RNA extractions from pOPC were performed using an identical protocol.

### 2.9 Gene expression analysis and NF‐yb knockdown

RNA samples were reverse transcribed using the Tetro cDNA synthesis kit (Bioline Reagents Ltd, London, UK) using the random hexamer primers provided. qPCR was performed using a Bio‐rad iQ5 PCR detection system with SYBR green PCR mastermix (Thermo Fisher Scientific, Paisley, UK, see Supporting Information Table S2 for qPCR primer sequences). Primer efficiency was tested using a 7‐point standard curve, and gene expression calculated using the 2^‐ΔΔCt^ method and normalized to the expression of β‐actin. For NF‐Yb knockdown, Oli‐neu cells were transfected with commercially available siRNA against NF‐Yb (siNF‐Yb, Qiagen Mm Nfyb1 FlexiTube siRNA SI01327207) or a negative control siRNA (siControl, Qiagen SI03650325). Knockdown of *NF‐*Yb in pOPC was achieved by transfection of a cocktail of four siRNA against *NF‐Yb* (siNF‐Yb multi, Qiagen FlexiTube Genesolution siRNA GS18045). For Olie‐neu cells siRNA constructs (5–50 nmol) were mixed with FuGENE HD (Promega Corporation, Madison, WI) at a ratio of 1:4 (µg cDNA:µl reagent), and cells transfected with the resulting complexes. *NF‐Yb* gene expression was examined 24 and 48 hr after transfection. In experiment using pOPC, siRNA constructs (40 nM) were mixed with Lipofectamine RNAiMAX (Thermo fisher Scientific). pOPC were incubated in the Liposome‐siRNA complexes for 6 hr, after which they were washed with OPC medium, and then cultured for a further 24 hr prior to use in experiments.

### 2.10 Western blot

Extracted proteins were separated by SDS‐Page using the Xcell sure lock mini‐cell system (Thermo Fisher Scientific), and then transferred onto PVDF membranes (Immobilion‐P, Merck Millipore, Watford, UK). Membranes were then incubated for 1 hr at room temperature in blocking medium (5% non‐fat milk dissolved in TBST [0.05 M Tris, 0.15 M NaCl, pH 7.2, 0.1% (v/v) Tween20]), before incubation in primary antibodies overnight at 4°C with constant agitation. After washing in TBST membranes were incubated in secondary antibodies for 1 hr at room temperature. All antibodies were diluted in blocking medium (see Supporting Information Table S1 for details of antibodies).

### 2.11 Chromatin immunoprecipitation (ChIP)

ChIP experiments quantifying NF‐Yb binding to the *Gria4* intronic regulatory region were performed in accordance with previously published methods (Weaver et al., 2004; Zhang et al., 2010). Briefly, Oli‐neu cells were plated at a density of 1 × 10^6^ cells in T25 flasks and allowed to adhere overnight. Duplicate flasks received the following treatment pairings: CTZ alone or AMPA/CTZ; Control Sato or AMPA_24h_; DMSO control or Garcinol. Following these treatments cells were fixed in 1% PFA at RT for 10 min before the addition of 0.125 M glycine. The medium was then removed and saved and cells washed in ice cold PBS before collection with cell scrapers in Farnham lysis buffer (plus protease inhibitors). The stored cell medium and collected cells were then combined and pelleted at 4°C, before sonication (Soniprep 150 plus, MSE, Ltd, London, UK, [1 × 10 s maximum power]), followed by centrifugation of the resulting suspension and further sonication of supernatants at 7 × 10 s/20 amp. Supernatants were de‐crosslinked and purified to determine consistent fragment sizes on a 1.5% agarose gel. Chromatin was immunoprecipitated with an antibody against NF‐Yb (Supporting Information Table S1) while antibodies against RNA POL and normal mouse IgG were used for positive and negative controls respectively. Samples were then prepared for DNA elution by hydrolysis of crosslinks and all input‐digested DNA and output DNA purified using the Qiaquick PCR purification kit (Qiagen). Ratios of output DNA:input DNA were determined by qPCR with the primers detailed in Supporting Information Table S2. All ChiP experiments were performed on triplicate samples derived from five independent Oli‐neu cell cultures except for Garcinol treated groups where triplicate samples were assessed in four independent cultures.

### 2.12 Luciferase assays

Reporter constructs containing either the 822 bp *Gria4* regulatory sequence (wt*Gria4*), or a version with NF‐Y binding site mutations (Δ*Gria4*, see Supporting Information Table S3 for details) were synthesized commercially (GeneWiz, LLC, South Plainfield, NJ) and inserted into pGL4.23[Luc2/minP] (Promega Corporation). Reporter constructs were transfected (FuGENE HD, Promega Corporation) into Oli‐neu cells seeded in opaque 96‐well plates at a density of 8 × 10^4^ cells/well. Transfections involved 235 ng/well of the normalizing vector pGL4.74[hRluc/TK] plus 290 ng/well of either wt*Gria4*, Δ*Gria4*, or the empty pGL4.23[Luc2/minP] vector. Reporter activity was initiated 24 hr later by the Promega DLR assay (Promega Corporation) and recorded on a luminometer (Centro LB 960, Bethold Technologies, Harpenden, UK).

### 2.13 Transcriptome analysis

Transcriptomic profiling was carried out on extracted RNA (Qiagen, Crawley, UK) after the quality of RNA was assessed using an ND‐1000 spectrophotometer (Nanodrop, Wilmington, DE) and qualified using an Agilent 2100 Bioanalyzer (Agilent Technologies, Santa Clara, CA). Agilent two color 60K Mouse microarrays were used to assess whole genome gene expression according to the manufacturer's instructions. Control samples were labelled with Cy3 (green) and treated samples were labelled with Cy5 (red). For background correction, the linear models for microarray data (LIMMA) package (Ritchie et al., 2015) were used within R 3.3.2 (RCoreTeam, 2016). Normalization within arrays was conducted using the “Loess” function and normalization between arrays was conducted using the “Aquantile” function (Smyth & Speed, 2003). The “lmscFit” function was used for separate channel analysis of two‐color microarray data (Smyth & Altman, 2013) using Benjamini‐Hochberg to adjust *p* values.

### 2.14 Analysis of gene expression network models

To derive an interactome model for AMPA/CTZ and Garcinol treated cells, differentially expressed genes were used as “seeds” and all known protein/protein interactions between the seeds and their inferred immediate neighbors were identified to generate a biological network using the BioGRID model of the human Interactome (3.4.145; Chatr‐Aryamontri et al., 2015). Network generation and processing was performed using Cytoscape 2.8.3 (Smoot, Ono, Ruscheinski, Wang, & Ideker, 2011). A hierarchy of interacting network modules within the interactome models was constructed using the ModuLand plugin for Cytoscape 2.8.3 (Kovacs, Palotai, Szalay, & Csermely, 2010; Szalay‐Bekő et al., 2012). The central core unit of each network module (metanode) was defined as the 10 most central genes. A list of the unique genes in each metanode was generated and used as a model of the functional core of the associated network for further comparison. The String database was used to assess the integrity and connectivity of gene modules (Szklarczyk et al., 2015).

### 2.15 Data analysis and statistics

Normal distributions were tested using an online Shapiro‐Wilks tool (Dittami, 2009) and when present two‐group comparisons were made by *t* tests, and multiple comparisons examined by one‐way ANOVA followed by Tukey's Honest Significance Difference post hoc tests. Mann‐Whitney *U* tests were used for two‐group comparisons when non‐normal distributions were detected. All comparisons were tested using the online tool VassarStats (Lowry, 2009). Potential regulators of *Gria4* were identified by screening all known *Gria4* interactions (Ingenuity Pathway Analysis, Qiagen, Redwood City, CA). A fixed value of *p*_α _< .05 for two‐tailed tests was the criterion for reliable differences between groups. One‐tailed tests were used when predictions were made possible by preceding results. Cited values are s ± *SEM*s.

## 3 RESULTS

### 3.1 Expression of AMPAR subunits in oli‐neu cells

**Figure 1:**
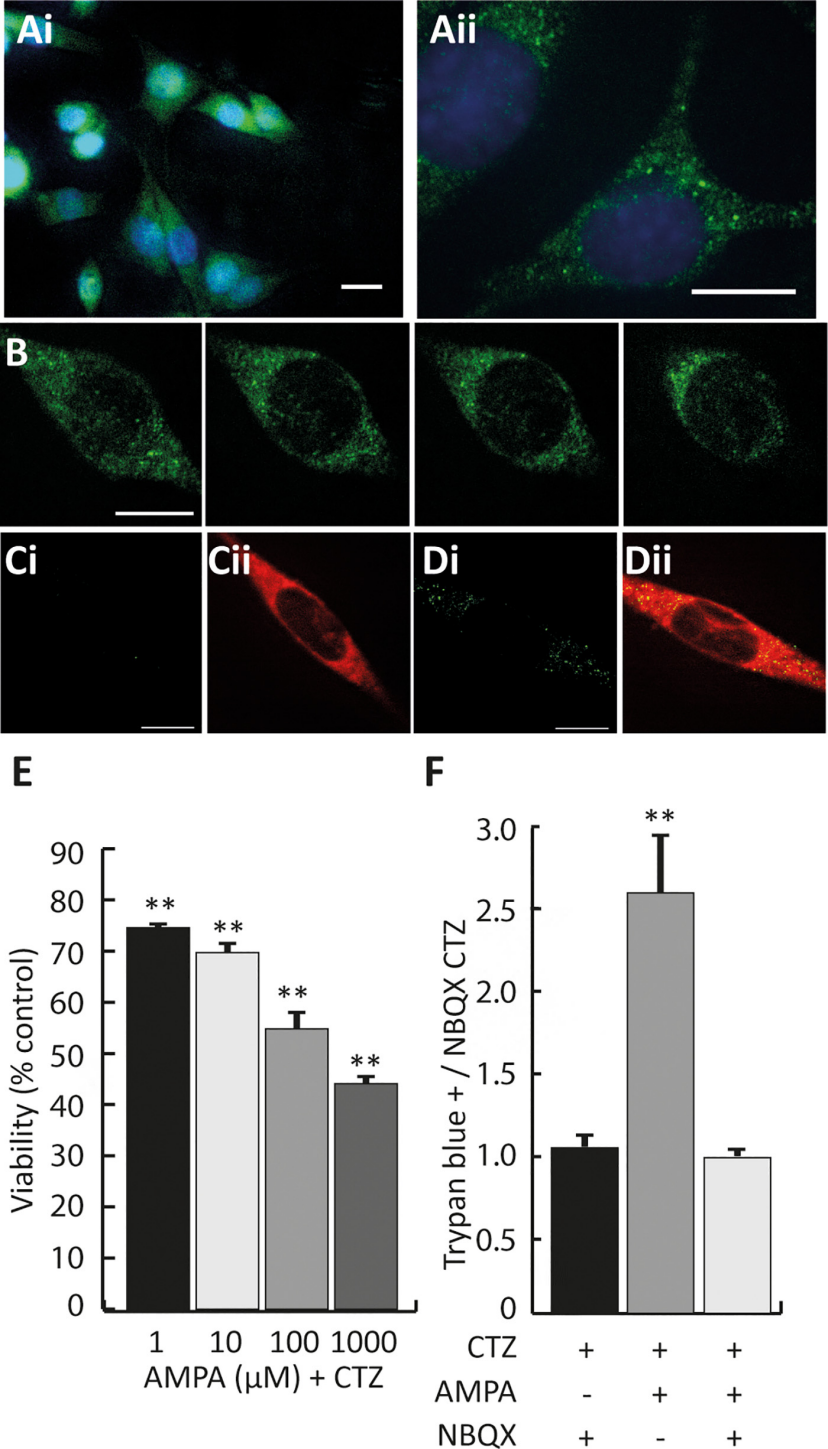
GluA4 expression and AMPAR‐mediated excitotoxicity in Oli‐neu cells. (a) Immunofluorescent labeling for GluA4 in Oli‐neu cells. (ai) Merged images for GluA4 (green), and DAPI (blue) at 20×. (aii) 100× image revealed punctate nature of GluA4 expression. (b) Sequences of confocal sections (100×; 0.2 µm) revealed GluA4 signal distribution consistent with plasma membrane localization (c) Membrane localization of GluA4. (ci) Faint immunofluorescent signal (green) from control sample incubated in secondary antibody alone. (cii) Merged image revealing no appreciable colocalization of the control secondary antibody stain with CellMask plasma membrane signals (red). (di) Distinct GluA4^+^ puncta (green) in a sample incubated with anti‐GluA4. (dii) Clear colocalization (yellow pixels) of GluA4 (green) and CellMask (red) signals in the cell depicted in di. (e) Prolonged activation of AMPAR caused a dose‐dependent reduction in Oli‐neu cell viability. Cell viability (% of viable cells normalized to DMSO treated controls) declined significantly after 5 hr exposure to 1 µM (74.6% ± 1.1%), 10 µM (69.7% ± 1.9%), 100 µM (54.8% ± 3.0%) and 1,000 µM (44.1% ± 1.5%) AMPA/CTZ. (f) NBQX prevented excitotoxicity in Oli‐neu cells. AMPA/CTZ induced an increase in Trypan blue^+^ cells (2.54 ± 0.35‐fold increase over NBQX/CTZ control) that was prevented by the AMPAR antagonist NBQX (0.93 ± 0.04‐fold increase over NBQX/CTZ control). Scale bars in a = 20 µm. Scale bars in b and c = 10 µm. ** Significance *p *<* *.01. Data expressed as means ± *SEM* [Color figure can be viewed at http://wileyonlinelibrary.com]

Oli‐neu cells are an established model for OPC (Jung et al., 1995; Pereira, Dobretsova, Hamdan, & Wight, 2011) previously used to study transcriptional regulation in oligodendroglia (Fratangeli et al., 2013). AMPAR expression has not been reported in Oli‐neu cells so we used qPCR and immunocytochemistry to detect AMPAR subunits GluA1–4. qPCR detected transcripts for all four GluA subunits (data not shown). Using immunofluorescent labeling GluA1 expression was limited to a few cells in which expression was restricted to the nucleus (Supporting Information Figure S1ai, S1aii). In contrast, GluA2, 3, and 4 were expressed by all cells (GluA2–3, Supporting Information Figure S1bi,ci; GluA4; Figure 1ai), with receptor subunits appearing in a punctate pattern throughout the soma and cell processes (GluA2–3, Supporting Information Figure S1bii,cii; GluA4, Figure 1aii). Although the permeabilizing conditions required for penetration of these antibodies precluded a definitive test of membrane expression, confocal optical sections revealed GluA4 signal distributions characteristic of membrane localization (Figure 1b). Further evidence for GluA4 membrane localization was provided by optical sections from cells labeled with the CellMask plasma membrane dye, where distinct GluA4 puncta colocalized with the membrane signal (Figure 1c,d). To confirm that AMPAR subunits form functional receptors in Oli‐neu cells we measured AMPAR‐mediated Ca^2+^ fluxes with the genetically encoded Ca^2+^ indicator GcAMP5. AMPA/CTZ induced small transient elevations in intracellular Ca^2+^ in ∼50% of the cells examined (Supporting Information Figure S2a). These data suggest that functional calcium‐permeable AMPAR are present at modest levels in Oli‐neu cells. We examined the ability of these receptors to mediate pathological signaling events by exposing Oli‐neu cells to AMPAR‐stimulating treatments likely to induce excitoxicity. Log concentrations of l‐glutamate (1–1,000 µM) applied for 5 hr reduced viability from 10 µM (Supporting Information Figure S2b; ANOVA *p *<* *.0001, control vs. 10 µM *p *<* *.05, control vs. 100 µM *p *<* *.01, control vs. 1,000 µM *p *<* *.01). We also examined viability after a 5‐hr exposure to AMPA/CTZ, which specifically and potently activates AMPAR without affecting other types of glutamate receptor. This treatment was more effective than l‐glutamate, with effects observed at the lowest concentration (1 µM; Figure 1e; ANOVA *p *<* *.0001, control vs. 1 µM *p *<* *.01, control vs. 10 µM *p *<* *.01, control vs. 100 µM *p *<* *.01, control vs. 1,000 µM *p *<* *.01). Importantly, the decrease in cell viability caused by AMPA/CTZ was blocked by pre‐incubation in the selective AMPAR antagonist NBQX (Figure 1f; ANOVA *p *<* *.01, AMPA/CTZ vs. CTZ + NBQX *p *<* *.01, AMPA/CTZ vs. AMPA/CTZ + NBQX *p *<* *.01). Prolonged stimulation with AMPA (200 µm) in the absence of CTZ inhibits OPC proliferation and differentiation without affecting viability (Yuan et al., 1998). We used this treatment, designated here as AMPA_24h,_ to determine if Oli‐neu cells are similarly sensitive to milder levels of AMPAR activation. AMPA_24h_ had no effect on Oli‐neu cell proliferation (Supporting Information Figure S3a; *p *=* *.83), or cell viability (Supporting Information Figure S3b; *p *=* *.38), showing that AMPA only affected Oli‐neu cells when co‐treated with CTZ. Incubation with 100 µm AMPA/CTZ produced a reliable decrease in cell viability (Figure 1e), so this treatment was chosen for studies aiming to uncover mechanisms regulating GluA4 transcription during excitotoxic injury.

### 3.2 Identifying transcriptional regulators of Gria4

An *in silico* approach designed to identify regulators of *Gria4* transcription highlighted NF‐Yb as a relevant target. *Gria4* was previously identified in a large‐scale screen of NF‐Yb binding sites carried out in HEPG2 cells (Testa et al., 2005). This information, together with its established role in controlling proliferation and cell death (Imbriano, Gnesutta, & Mantovani, 2012), actions that are regulated in oligodendroglia by AMPAR, marked NF‐Yb as a promising candidate for further study. The human *Gria*4 gene was screened for locations showing evidence of NF‐Y binding. This search identified a candidate region (chr11:105685481–105685873) in intron 4 where there was ChIP‐seq evidence from the ENCODE data analysis of the GM12878 cell line (a lymphoblastoid line) that supported NF‐Yb interactions with the DNA. There was also evidence at the same location for the histone marks H3K4Me1, often found near regulatory elements, and H3K27Ac, often found near active regulatory elements, in GM12878 and a range of other ENCODE cell lines. The identified region corresponded to chr9:4,601,105‐4,601,164 in intron 4 of the mouse *Gria4* gene. A search for putative NF‐Yb binding sites (CCAAT) within this region of the mouse genome identified three such sequences (termed sites 1–3 in this article, Supporting Information Table S2). This region is located 3′ to the transcription start site (TSS) and so falls outside of the classic proximal promoter position associated with CCAAT elements (Mantovani, 1999).

### 3.3 Excitotoxic AMPAR stimulation decreased expression of NF‐yb and GluA4

**Figure 2:**
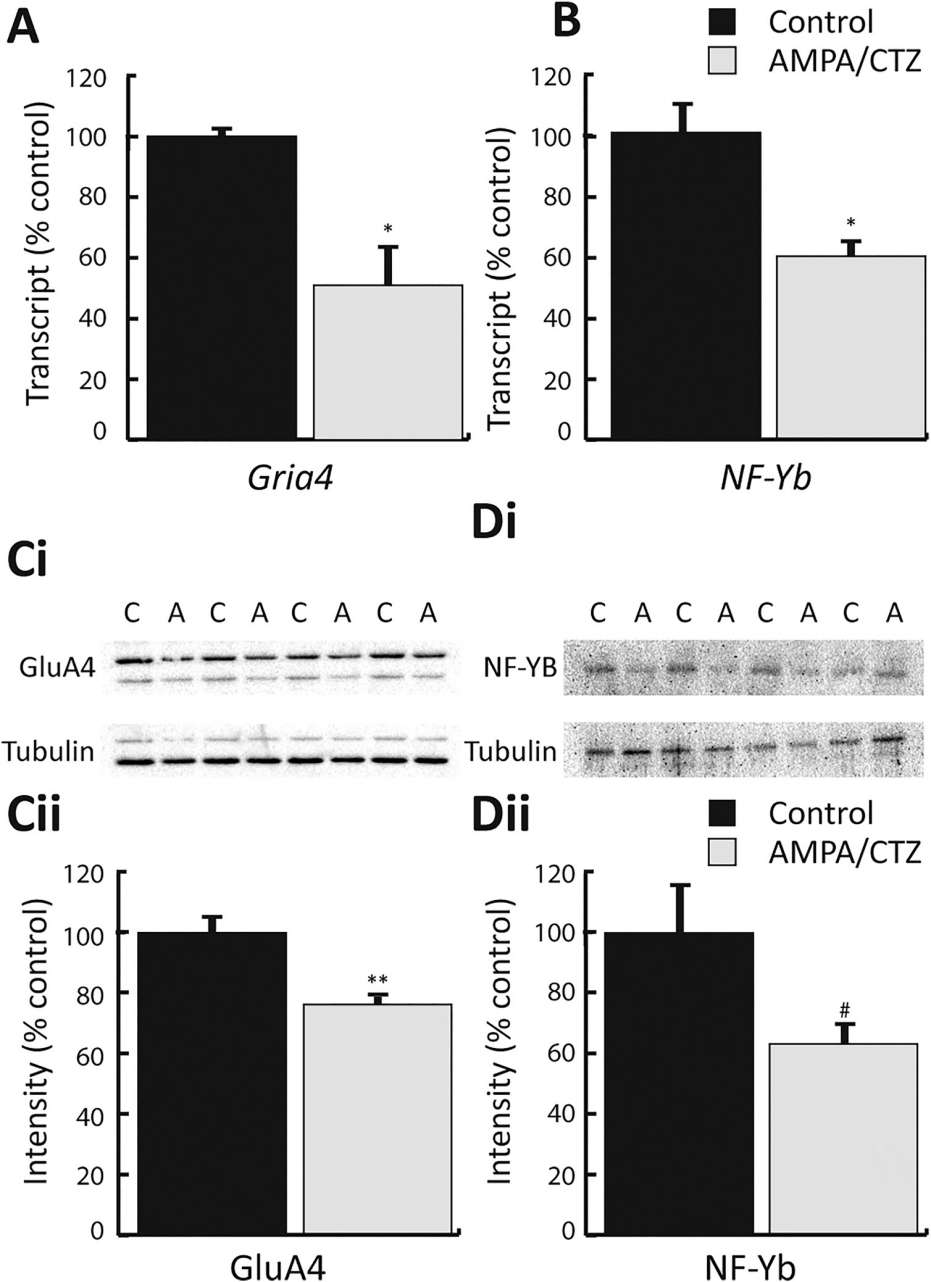
Treatment with AMPA/CTZ decreased expression of GluA4 and NF‐Yb. (a and b) Transcript levels for *Gria4* (a) and *NF‐Yb* (b) were significantly reduced by a 5‐hr exposure to AMPA/CTZ (*Gria4*: Control 1.00 ± 0.03, AMPA 0.51 ± 0.15; *NF‐Yb*: Control 1.01 ± 0.11, AMPA 0.60 ± 0.06). (c and d) Western blot analysis from Oli‐neu cells treated with AMPA/CTZ. (ci and di) Representative immunoblots for GluA4 (ci), NF‐Yb (di) and the loading control protein α‐tubulin from four independent experiments. (cii and dii) Densitometric analysis of immunoblots shown above (normalized against α‐tubulin) indicated reduced levels of GluA4 (cii) and NF‐Yb (dii) protein after exposure to AMPA/CTZ (GluA4: Control 1.00 ± 0.05, AMPA 0.76 ± 0.04; NF‐Yb: Control 1.00 ± 0.12, AMPA 0.69 ± 0.08). * and ** Significance *p *<* *.05 and *p *<* *.01, respectively, # significance *p *<* *.05 one‐tailed. Data expressed as means ± *SEM*

Prolonged activation of AMPAR in pOPC cultures has been shown to induce alterations in the expression of GluA4 protein (Hossain et al., 2014). To probe possible mechanisms regulating *Gria*4 transcription, we measured *Gria4* and *NF‐Yb* transcript and protein in Oli‐neu cells subjected to AMPA_24h_. Similar to our observations of cell viability and proliferation, transcription of *Gria4* and *NF‐Yb* remained unaffected by AMPA_24h_ (Supporting Information Figure S4a,b; *Gria4*, *p *=* *.98; *NF*‐*Yb, p *=* *0.64). Levels of GluA4 (Supporting Information Figure S4ci,cii) and NF‐Yb (Supporting Information Figure S4ci,ciii) protein were also unaffected by this AMPA treatment (GluA4, *p *=* *.75; NF‐Yb, *p *=* *.86). Given the modest extent of functional AMAPR expression in Oli‐neu cells we considered whether stronger AMPAR activation might be required to modulate GluA4 expression. Indeed, treatment of Oli‐neu with AMPA/CTZ for 5 hr led to a significant reduction in the level of *Gria4* mRNA (Figure 2a; *p *<* *.05) and GluA4 protein (Figure 2ci,cii; *p *<* *.001). Treatment with AMPA/CTZ also reduced the levels of NF‐Yb mRNA and protein (Figure 2b,di,dii) (NF‐Yb mRNA, *p *<* *.05; NF‐Yb protein, *p *<* *.04), suggesting that levels of *Gria4* expression may be related to those of *NF‐Yb*.

### 3.4 Excitotoxic AMPAR stimulation decreased enrichment of NF‐yb at Gria4 binding sites

**Figure 3:**
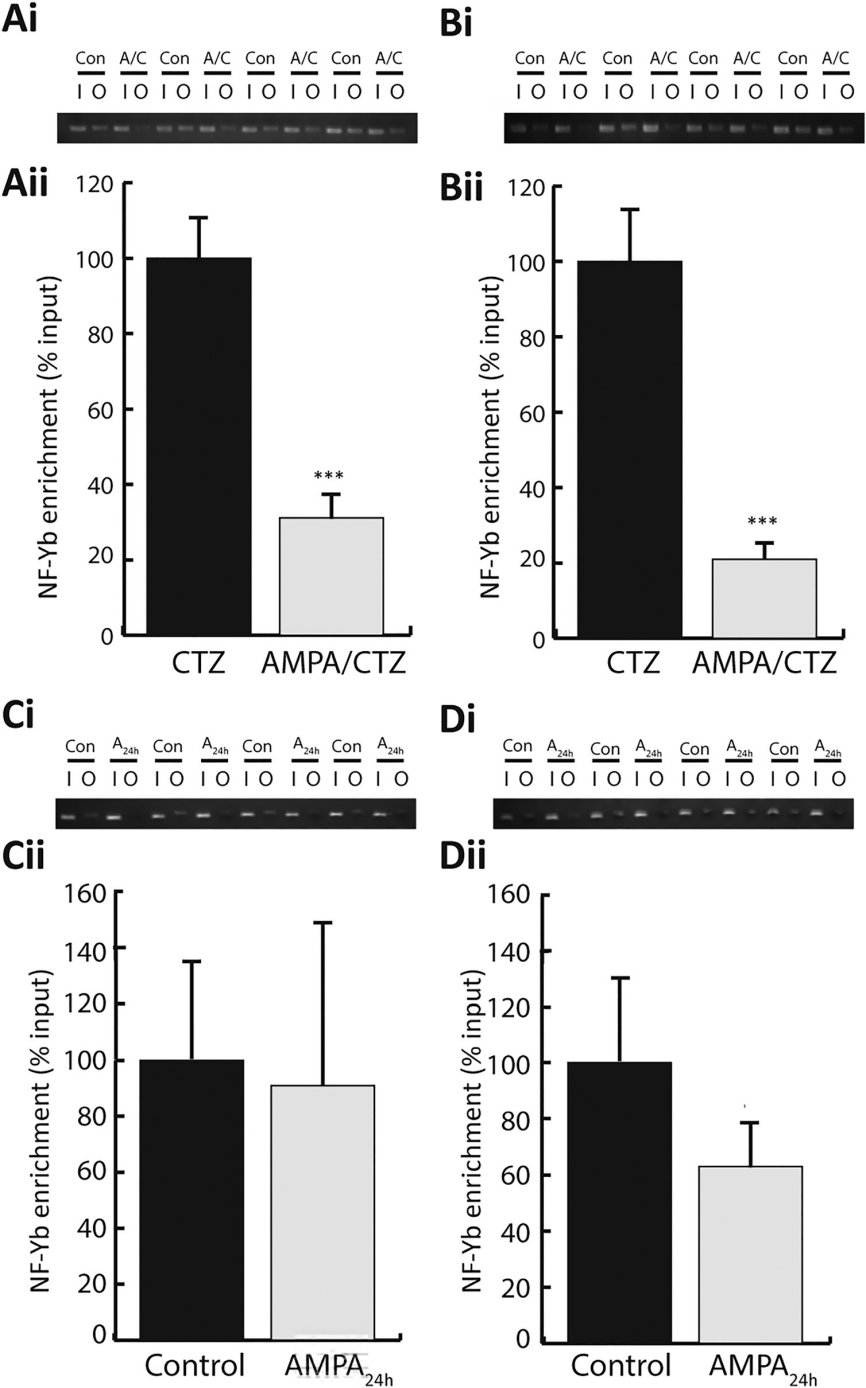
Treatment with AMPA/CTZ but not AMPA_24h_ altered binding of NF‐Y to *Gria*4. (a and b) ChIP assays indicated a significant reduction in enrichment of NF‐Yb at CCAAT sites 1 (a) and 2 (b) after treatment with AMPA/CTZ. (ai and bi) show RT‐PCR gels for four independent experiments on sites 1 and 2, respectively. I, input signal; O, output signal; Con, control; A/C, AMPA/CTZ. (aii and bii) Quantification of NF‐Yb enrichment at sites 1 and 2, respectively (normalized to control average). Treatment with AMPA/CTZ significantly reduced binding of NF‐Yb at site 1 (CTZ control 1.00 ± 0.12, AMPA/CTZ 0.31 ± 0.07) and site 2 (CTZ control 1.00 ± 0.14, AMPA/CTZ 0.21 ± 0.04). (c and d) ChIP assays showing unaltered levels of NF‐Yb at CCAAT sites 1 (c) and 2 (d) on the *Gria*4 promoter after AMPA_24h_. (ci and di) show RT‐PCR gels for four independent experiments on sites 1 and 2, respectively. I, input signal; O, output signal; Con, control; A_24h_, AMPA_24h_. (cii and dii) Quantification of NF‐Yb enrichment at site 1 (normalized to control average) (CTZ control 1.00 ± 0.35, AMPA/CTZ 0.92 ± 0.59) and site 2 (CTZ control 1.00 ± 0.30, AMPA/CTZ 0.63 ± 0.16). *** Significance *p *<* *.0001. Data expressed as means ± *SEM*

The corresponding changes in *NF‐Yb* and *Gria4* detected after AMPA/CTZ (Figure 2) may indicate that both genes are targets of the same regulator, or that NF‐Yb directly regulates *Gria4*. We performed ChiP assays directed at CCAAT sites 1–3 (Supporting Information Table S3) to determine whether NF‐Yb physically interacted with these CCAAT sites, and if binding was modulated by excitotoxic injury. Treatment with AMPA/CTZ resulted in a significant reduction in NF‐Yb enrichment at sites 1 (*p *<* *.0001; Figure 3a) and 2 (*p *<* *.01; Figure 3b) but not site 3 (data not shown). In agreement with our analysis of *Gria*4 transcription (Supporting Information Figure S3), AMPA_24h_ treatment had no effect on NF‐Yb binding (sites 1 and 2; Figure 3c,d; site 3 data not shown; site 1, *p *=* *.63; site 2, *p *=* *.31). The observed decrease in *Gria4* transcription following AMPA/CTZ (Figure 2a), and the ChIP data, suggested that NF‐Yb‐*Gria4* interactions play an important role in the regulation of *Gria4* transcription.

### 3.5 Pharmacological inhibition of NF‐Y reduced NF‐yb function and triggers apoptotic cell death

**Figure 4:**
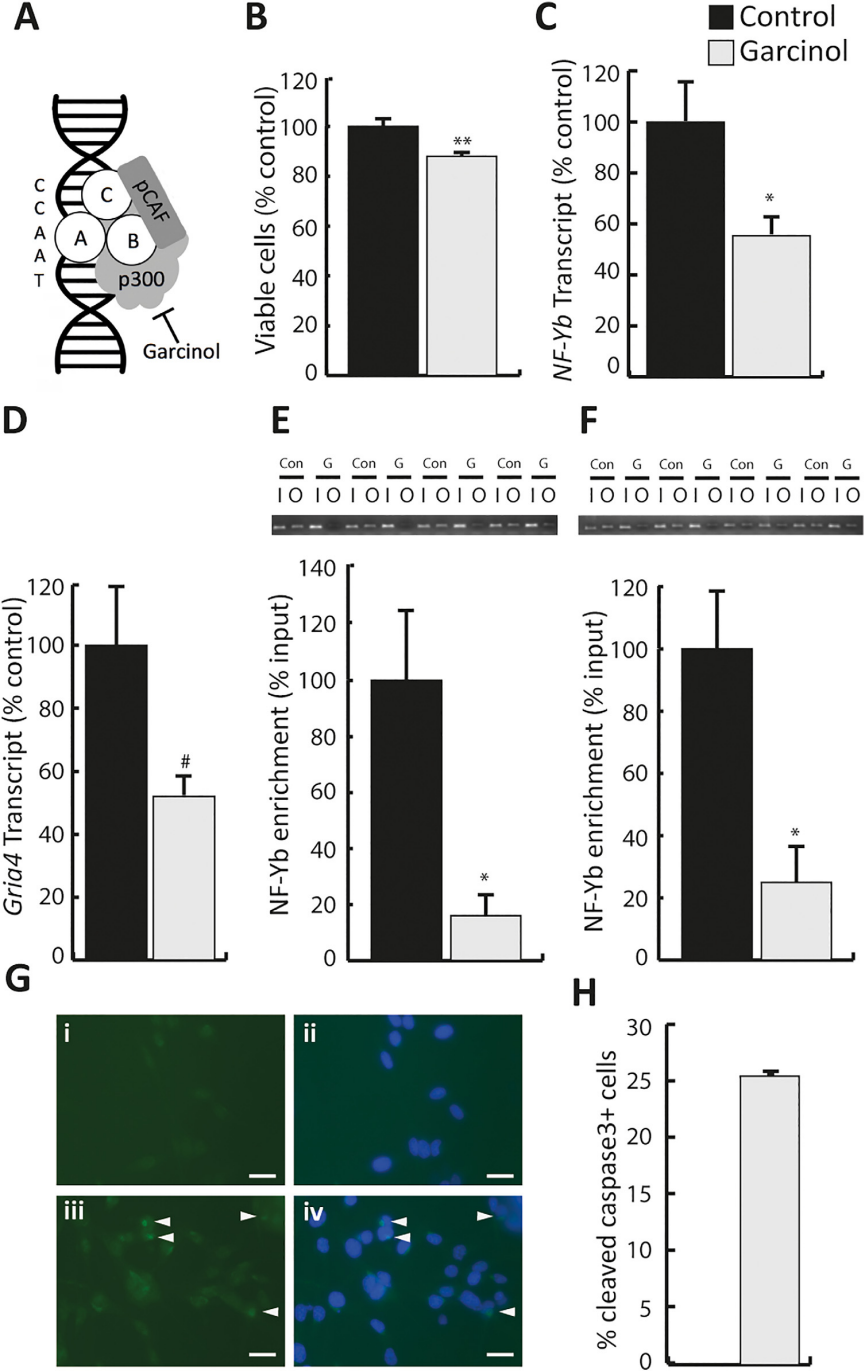
Garcinol reduced cell viability, NF‐Yb transcription and NF‐Yb *Gria4* interactions. (a) Model illustrating organization of the NF‐Y complex, it's association with CCAAT sites, and regulation through Garcinol sensitive p300 (Faniello et al., 1999). (b) Garcinol reduces Oli‐neu cell viability. Cell viability normalized to the control treatment was reduced following a 5‐hr treatment with Garcinol (10 µM; control 1.00 ± 0.02, Garcinol 0.88 ± 0.01). (c) Transcript levels for NF‐Yb were significantly reduced by a 5‐hr exposure to Garcinol (control 1.00 ± 0.15, Garcinol 0.55 ± 0.06). (d) Garcinol reduced levels of GluA4 transcripts (control 1.00 ± 0.19, Garcinol 0.51 ± 0.06). (e and f) Treatment with Garcinol disrupted binding of NF‐Y to the *Gria*4 promoter. Top panels show RT‐PCR gels for four independent experiments on sites 1 (e) and 2 (f). I, input signal; O, output signal; Con, control; G, Garcinol. Lower panels show quantification of NF‐Yb enrichment at sites 1(e) and 2 (e). Enrichment of NF‐Yb at both sites was significantly reduced following treatment with Garcinol at site 1 (control 1.00 ± 0.25, Garcinol 0.16 ± 0.08) and site 2 (control 1.00 ± 0.19, Garcinol 0.25 ± 0.12). (g) Representative cleaved Caspase‐3^+^ immunostaining (green) with DAPI (blue) in Oli‐neu cells after 24‐hr treatment with DMSO vehicle (gi and gii) and Garcinol (giii and giv). White arrowheads indicate locations with cleaved Caspase3^+^ immunofluorescence within the nucleus indicative of apoptosis. (h) Cleaved caspase‐3^+^ was undetectable in control treated cells, but was observed in 25% of cells after Garcinol treatment. * and ** Significance *p *<* *.05 and *p *<* *.01 respectively, # significance *p *<* *.05 one‐tailed. Scale bars in f 50 µm. Data expressed as means ± *SEM* [Color figure can be viewed at http://wileyonlinelibrary.com]

The data presented above indicate NF‐Y as a regulator of *Gria4* transcription during excitotoxic injury. Further support for this idea was sought by performing pharmacological experiments to disrupt NF‐Y activity in Oli‐neu cells. NF‐Y function depends on interactions between NF‐Yb and the nuclear co‐activator protein p300 (Figure 4a; Faniello et al., 1999). Therefore, we used the p300 inhibitor Garcinol (Balasubramanyam et al., 2004) to examine the impact of reduced NF‐Y function on *Gria4* transcription. The effect of Garcinol on oligodendroglia has not been reported previously. As described in Materials and Methods concentrations of Garcinol >10 µM produced widespread cell death with the majority of cells detaching from the dish within 5 hr of application. At 10 µM the majority of Oli‐neu remained attached to the dish, and a Trypan blue assay indicated a significant reduction in Oli‐neu viability (Figure 4b; *p *<* *.01). Moreover, this concentration of Garcinol also reduced transcription of *NF‐Yb* (Figure 4c; *p*_ _< .05) and *Gria4* (Figure 4d; *p *<* *.05). To test if reduced NF‐Y function associated with alterations in *Gria4* expression, we examined enrichment of NF‐Yb at *Gria4* CAAT sites 1–3 following Garcinol treatment. Garcinol decreased NF‐Yb binding to at CAAT sites 1 and 2 (Figure 4e,f; site 1, *p *<* *.05; site 2, *p *<* *.05), but not 3 (data not shown). These data confirm that Garcinol induced the same changes in NF‐Yb/*Gria4* interactions, and *Gria4* transcription to those observed after AMPAR‐mediated excitotoxicity. To determine if disruption of NF‐Ywas associated with apoptosis, we examined nuclear labeling for cleaved caspase‐3 following a 5‐hr Garcinol treatment. 24 hr after this treatment cleaved caspase‐3 was undetectable in vehicle treated cells (Figure 4gi,gii), but could be observed in the cytoplasm and nuclei of Oli‐neu cells exposed to Garcinol (Figure 4giii,giv). The % of cleaved caspase‐3^+^ cells was 25% (Figure 4h), suggesting caspase‐3‐dependent apoptosis.

### 3.6 NF‐yb knockdown reduced transcription of constructs containing the Gria4 regulatory region

**Figure 5:**
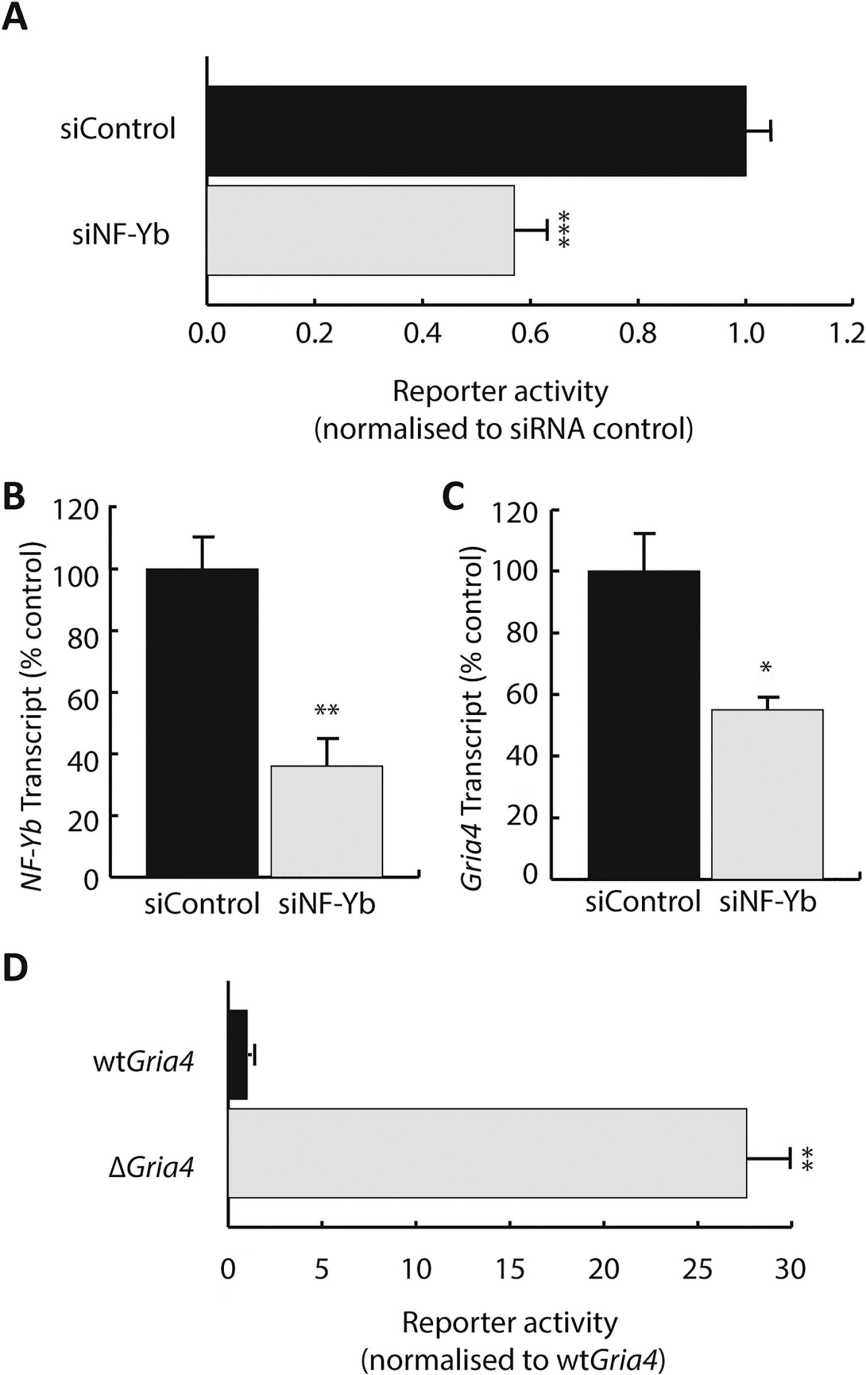
Knockdown of NF‐Yb altered the influence of *Gria4* regulatory region and *Gria4* transcription. (a) Activity of a reporter construct harboring wt*Gria4* NF‐Y sites (normalized to control averages) from Oli‐neu cells transfected with siControl and siNF‐Yb. Reporter activity was reduced in cells transfected with siNF‐Yb (siControl 1.00 ± 0.05, siNF‐Yb 0.57 ± 0.06). (b and c) Transcript levels (normalized to control averages) for *NF‐Yb* (b) and *Gria4* (c) were significantly reduced in cells transfected with siNF‐Yb (NF‐Yb: siControl 1.00 ± 0.11, siNF‐Yb 0.36 ± 0.09; GluA4: siControl 1.00 ± 0.12, siNF‐Yb 0.55 ± 0.04). (d) Luciferase reporter activity (normalized to control averages) from Oli‐neu cells transfected with wt*Gria4* and Δ*Gria4* constructs. Reporter activity (normalized to wtGluA4 control average) was significantly enhanced by mutations of the NF‐Y sites (wtGluA4 1.00 ± 0.08, ΔGluA4 27.62 ± 2.33). *, **, *** Significance *p *<* *.05, *p *<* *.01 and *p *<* *.001 respectively. Data are expressed as means ± *SEM*

To test the influence of NF‐Y on the CCAAT‐containing regulatory region we performed reporter assays on Oli‐neu cells transfected with the wt*Gria4* construct (Supporting Information Table S3) and either siNF‐Yb or siControl. Knockdown of *NF‐Yb* reduced reporter activity from the wt*Gria4* construct by 54% relative to siControl transfected cells (Figure 5a; *p *<* *.0001). To verify the efficiency of the knockdown, we measured *NF‐Yb* transcripts 24 hr after transfection with siNF‐Yb. Oli‐neu cells transfected with siNF‐Yb displayed a 64% reduction in *NF‐Yb* mRNA compared with cells transfected with siControl (Figure 5b; *p *<* *.01). *NF‐Yb* knockdown also reduced the level of *Gria4* transcription (Figure 5c; *p *<* *.05) further supporting a role for NF‐Y in the control of *Gria4* expression. We sought additional evidence for the involvement of NF‐Y in *Gria4* transcription by generating a reporter construct containing mutations in NF‐Yb binding sites 1–3 (Δ*Gria4*, Supporting Information Table S3). In contrast to NF‐Yb knockdown, cells transfected with Δ*Gria4* exhibited a 27.6‐fold increase in activity of the reporter compared with cells treated with the wtGluA4 construct (Figure 5d; *p *<* *.01).

### 3.7 NF‐yb regulates Gria4 expression and cell survival in primary OPC

**Figure 6:**
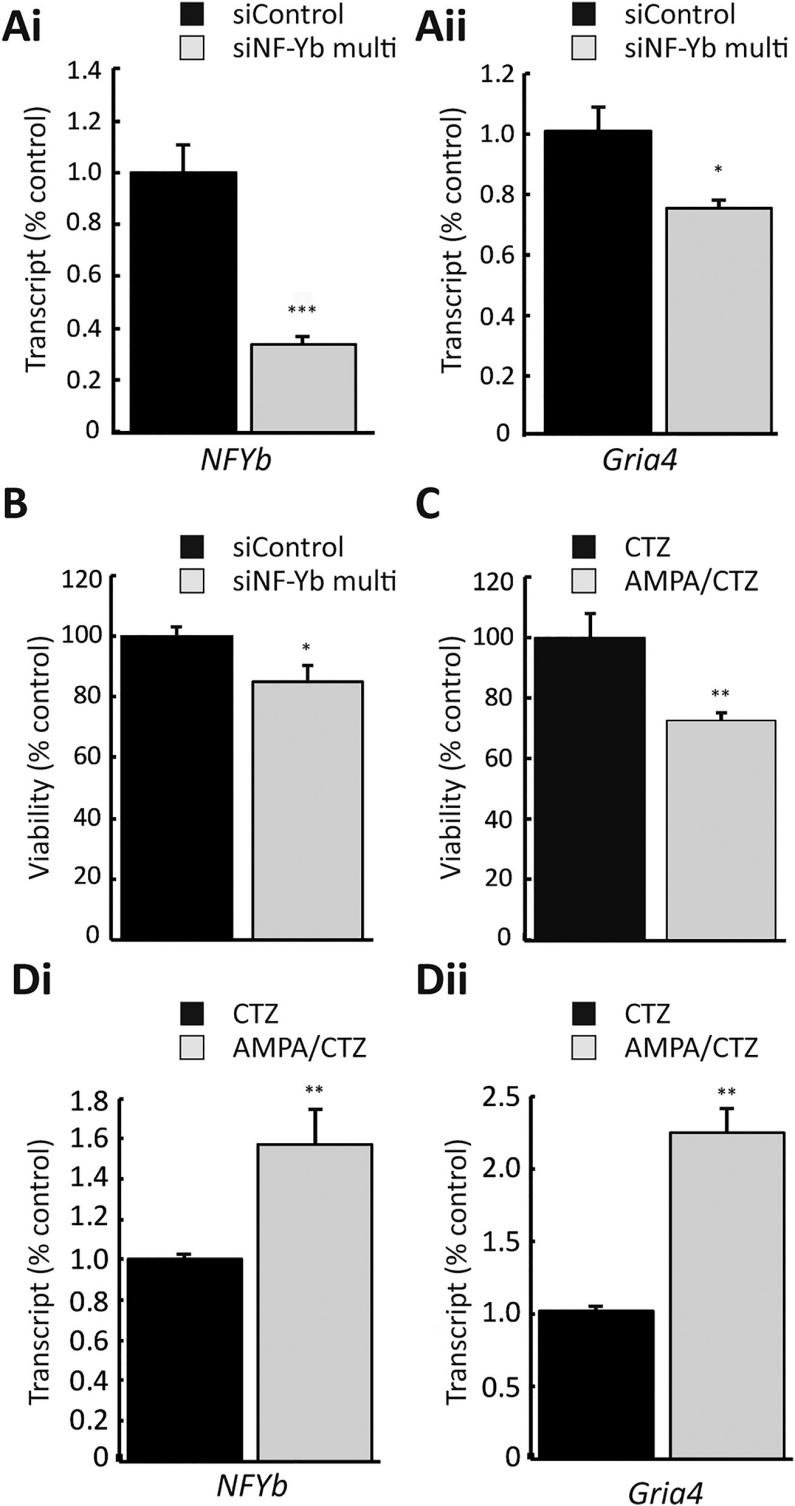
*NF‐Yb* regulates *Gria*4 transcription and cell survival in primary OPC. (ai) Transfection of pOPC with a cocktail of four siRNA targeted against *NF‐Yb* produces a significant reduction in *NF‐Yb* mRNA (siControl 1.00 ± 0.1, siNF‐Yb multi 0.34 ± 0.02). (aii) Knockdown of *NF‐Yb* leads to a significant reduction in *Gria*4 mRNA relative to siControl levels (siControl 1.00 ± 0.07, siNF‐Yb multi 0.75 ± 0.02). (b) Knockdown of *NF‐Yb* reduces pOPC cell viability. pOPC viability normalized to siControl values was reduced 24 hr after transfection with siNF‐Yb multi (siControl 100 ± 2.73, siNF‐Yb multi 84.99 ± 5.01). (c) Excitotoxic treatment compromises the viability of pOPC. The proportion of viable pOPC (normalized to CTZ control) is significantly reduced after 5 hr of AMPA/CTZ (CTZ control 100 ± 2.00, AMPA/CTZ 78.25 ± 2.33). (di and dii) Excitotoxic stimulation increases transcription of *NF‐Yb* and *Gria4* relative to CTZ control levels (*NF‐Yb*: CTZ control 1.00 ± 0.01, AMPA/CTZ 1.57 ± 0.10; *Gria4*: CTZ control 1.03 ± 0.10, AMPA/CTZ 2.25 ± 0.17). *, **, ** Significance *p *<* *.05, *p *<* *.01, and *p *<* *.001, respectively. Data are expressed as means ± *SEM*

Experiments were performed to determine if the molecular and cellular results obtained in Oli‐neu cells reflect the role of NF‐Yb in pOPC. In agreement with the data from Oli‐neu cells, knockdown of *NF‐Yb* (Figure 6ai) produced a significant reduction in levels of *Gria4* (Figure 6aii; *p *<* *.05). Similarly, *NF‐Yb* knockdown was associated with a reduction in OPC viability (Figure 6b; *p *<* *.05) that mirrored the effect observed following pharmacological inhibition of *NF‐Yb* by Garcinol (Figure 4b). Treatment with AMPA/CTZ also reduced the viability of pOPC (Figure 6c; *p *<* *.05). However, in pOPC excitotoxic injury produced an increase in the levels of both *NF‐Yb* (Figure 6di; *p *<* *.01) and *Gria4* (Figure 6dii; *p *<* *.01) mRNA. Thus, while the relationship between *NF‐Yb* and *Gria*4 was maintained, the direction of change in pOPC opposed that observed in Oli‐neu cells.

### 3.8 Transcriptomic analysis indicated similar effects of AMPA/CTZ and garcinol on apoptotic genes

**Figure 7:**
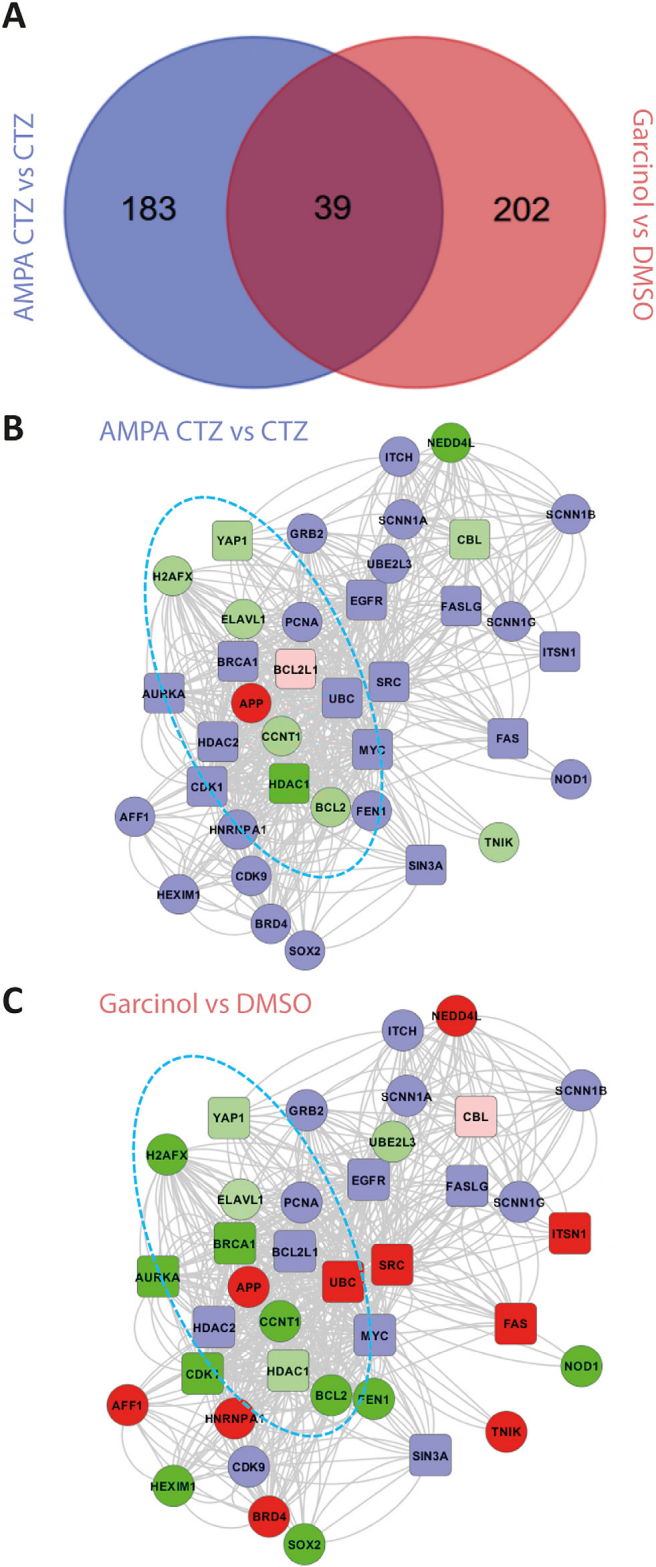
Interactome modelling from AMPA/CTZ and Garcinol treatments identified a core network element with consistent differential gene expression. (a) Overlap of the central network hierarchies identified by the moduland algorithm (numbers of genes). A network of the common 39 differentially regulated genes was defined using the STRING database (confidence > 0.4) in Oli‐neu cells treated with AMPA/CTZ (b) and Garcinol (c). Blue = inferred interaction; green = down regulated; red = up regulated gene expression with Oli‐neu cell treatments. Shade of green/red indicate level of fold change. Rounded squares = genes associated with control of apoptosis. All differential gene expression *p *<* *.01 [Color figure can be viewed at http://wileyonlinelibrary.com]

Our RNAi and pharmacological data suggest a role for NF‐Y in the regulation of OPC survival. This conclusion agrees with findings from other cell types regarding the role of NF‐Y in the regulation of apoptosis and viability (Benatti et al., 2008; Jiang et al. 2015; Morachis et al. 2010; Mojsin et al. 2015). To explore the wider context of the downstream functions associated with NF‐Y in oligodendroglia we performed a transcriptomic study in Oli‐neu cells subjected to excitotoxic injury and pharmacological disruption of NF‐Y. The top 500 differentially expressed genes ranked by *p* value (*p < *.001) from the two injury treatments (AMPA/CTZ and Garcinol) were used to generate interactome models (Supporting Information Tables S4 and S5). The central hierarchy of each interactome model (top 30 gene modules) was determined using the Moduland algorithm (Supporting Information Tables S6 and S7). Comparison of central interactome hierarchies identified 39 common genes (*p < *.0001; Figure 7a). Control of apoptosis was associated with 17 of these 39 genes (*p = *1.1 × 10^−10^; rounded squares in Figure 7b,c – Supporting Information Table S8). The shared network of 39 genes was more connected than expected by chance (*p *<* *.001) and a central element was identified (dashed blue ellipse in Figure 7b,c) in which treatments induced identical differential gene expression.

## 4 DISCUSSION

In this work, we used excitotoxic levels of AMPAR stimulation to uncover mechanisms regulating the expression of GluA4 in cells of the oligodendrocyte lineage. We identified an intronic regulatory region harboring binding sites for NF‐Yb, whose binding by this transcription factor are down‐regulated during excitotoxic injury. Decreased NF‐Yb binding is accompanied by a reduction in the levels of GluA4 mRNA and protein, an effect mimicked at the transcriptional level by siRNA knockdown of *NF‐Yb*. We also show that reducing NF‐Y function through pharmacological inhibition and knockdown compromises the survival of Oli‐neu and pOPC respectively, and that reduced NF‐Y function alters the expression of a number of apoptotic genes. Together, these data provide the first example of a *trans‐acting* mechanism controlling *Gria4* expression, and highlight the NF‐Y pathway as a regulator of OPC viability and survival.

### 4.1 Studying transcriptional regulation of AMPAR in oli‐neu cells

Oli‐neu cells have been used to study aspects of oligodendrocyte biology including myelin protein trafficking (Dhaunchak, Colman, & Nave, 2011; Trajkovic et al., 2006), exosome release (Frühbeis et al., 2013), and the expression and function of connexins (Sohl, Hombach, Degen, & Odermatt, 2013) and G protein coupled receptors (Fratangeli et al., 2013; Simon et al., 2016). The ease of generating large, high purity, cultures of these cells, and their ease of transfection relative to pOPC, make them particularly useful for studies of transcript expression and regulation where large numbers of homogenous cells must be manipulated and harvested for mRNA and protein sampling (Gobert et al., 2009; Iacobas & Iacobas, 2010; Joubert et al., 2010). In this regard, their utility as a model for OPC biology is further supported by data from the present study showing that alterations in NF‐Y function induce produces similar effects on *Gria4* transcription and cell viability in both Olie‐neu and pOPC.

The expression and regulation of glutamate receptors has not been explored in Oli‐neu cells, although this was examined in electrophysiological studies of the closely related cb‐neu cell line (Jung et al., 1995), in which the absence of membrane currents was reported following exogenous application of glutamate. In contrast, the present paper provides two independent lines of evidence (Ca^2+^ imaging and excitotoxic cell death) that suggest modest levels of functional AMPAR expression in Oli‐neu. Despite these low levels of AMPAR function, Oli‐neu cells proved useful for detecting excitotoxic changes in GluA4 subunit expression and the transcriptional machinery controlling these processes.

### 4.2 Excitotoxicity‐induced changes in Gria4 transcription in olie‐neu and pOPC

Depending on the cell type examined, excitotoxic injury either decreased (Oli‐neu) or increased (pOPC) transcription of *Gria4*. These data from pOPC reflect those from an *in vivo* study of GluA4 expression in neonatal rats exposed to hypoxia (Sivakumar, Ling, Lu, & Kaur, 2010). Here, exposure to hypoxia that induces strong elevations in the concentration of extracellular glutamate is associated with a significant increase in GluA4 immunoreactivity within immature oligodendroglia. The expression of *Gria4* is regulated developmentally (Itoh et al., 2002; Hossain et al., 2014), thus the direction of excitotoxicity‐induced regulation of *Gria4* in oligodendroglia may depend on maturational status. Indeed, Oli‐neu cells cultured under the conditions used in the present study exhibit low levels of immunoreactivity to anti‐O4 (Toutouna et al., 2016), suggesting that this cell line holds a more immature position in the lineage than pOPC, which typically exhibit strong anti‐04 staining. This difference not withstanding, other aspects of the relationship between NF‐Yb, *Gria4* transcription and cell viability were validated in pOPC, supporting a role for NF‐Yb in the regulation of *Gria4* and cell survival in OPC.

### 4.3 Characteristics of the intronic CCAAT regulatory region in mouse Gria4

The CCAAT box is a common promoter element occupying a position typically located between −60 and −100 from the TSS (Mantovani, 1999), where it exerts well‐described actions on promoter activity (Dolfini, Gatta, & Mantovani, 2012). Prior to the present work NF‐Y had not been studied in oligodendroglia. However, CCAAT sites located in the promoter of protein zero, a key myelin gene in the peripheral nervous system, regulate protein zero transcription in Schwann cells (Brown & Lemke, 1997). The CCAAT sites identified in the present paper are located 3′ to the *Gria4* TSS so fall outside of the proximal promoter region. These observations add to a growing body of evidence demonstrating roles for NF‐Y outside of classic promoter regions. For example, a recent genome‐wide mapping study revealed that 25% of NF‐Y peaks are associated with distal enhancer regions (Fleming et al., 2013). In addition, expression arrays performed following NF‐Ya inactivation revealed that 50% of differentially expressed genes exhibit NF‐Y/DNA binding outside of promoter regions. The importance of NF‐Y/enhancer interactions have also been validated i*n vivo*, where they are found to regulate tissue specific expression of *Hoxb4* during embryonic development (Gilthorpe et al., 2002).

Excitotoxic injury reduced NF‐Yb binding to CCAAT sites located within the *Gria4* intronic regulatory region and produced a parallel reduction in the level of *Gria4* transcripts. In agreement with this, our functional studies using a reporter construct containing the *Gria4* regulatory regions showed that knockdown of *NF‐Yb* reduced activity of a minimal promoter. Overall these data from qPCR, ChiP and reporter assays are consistent with a role for NF‐Y in regulating *Gria4* transcription in Oli‐neu cells. We also examined NF‐Y actions at these sites using a reporter construct bearing mutations designed to prevent NF‐Y binding. Surprisingly, these mutations led to a significant increase in reporter activity. Knockdown of *NF‐Yb* had no effect on promoter activity when the *Gria*4 region was absent. Thus, the opposing actions of the NF‐Y site mutations and siRNA knockdown, cannot be explained on the basis of NF‐Y actions on sequences located outside of the regulatory region. These results could be explained if mutation of the binding sites hindered the actions of another repressive *trans‐acting* factor that exerts actions on the promoter *via* the CCAAT elements. Indeed, the high abundance of H3K27Ac marks in this region of *Gria4* suggests it is highly regulated. One possible candidate is the CCAAT/enhancer binding proteins (C/EBPs), which have a well described ability to bind CCAAT boxes (Landschulz, Johnson, Adashi, Graves, & McKnight, 1988). Irrespective of these possibilities, the knockdown, qPCR and ChIP experiments presented in this paper highlight NF‐Yb as an effective regulator of *Gria*4 transcription.

### 4.4 First trans‐acting mechanism regulating GluA4: Implications for protective therapies

To our knowledge, the actions of NF‐Yb reported in the present study describe the first *trans‐acting* mechanism controlling GluA4 expression. Earlier work examining the promoter of rat *Gria4* described two potential regulatory regions (Borges, Myers, Zhang, & Dingledine, 2003). The first was a long interspersed element (LINE) located 3.6–3.9 kb upstream from the translation start site (TSS), which was found to contain silencing elements. This work also identified several TSS in Rat *Gria4* located between −1,090 and −1,011. Importantly, the LINE silencing region is distant from these sites being ∼2–3 kb further upstream, and so contrasts greatly with the regulatory region we have described in mouse *Gria4*, which is located 3′ of the TSS. The second regulatory region in Rat *Gria4* contained a cluster of transcription factor sites (e.g., SP1, CAAT box, IK2, MRE) located close to the TSS (Borges et al., 2003). Although the authors did not determine the influence of these sites on transcription directly, they suggested their involvement in directing neuronal selectivity since deletion of the 177 bp region containing them reduced transcription of *Gria4* in neuronal, but not glial cultures.

New data from the present study indicate that NF‐Y plays an important role in the response of oligodendroglia to cellular stress. Both excitotoxic injury and a pro‐apoptotic treatment with Garcinol initiated a change in NF‐Y function that was tightly linked to alterations in *Gria4* transcription. Reduced levels of GluA4 could protect oligodendrocytes from further injury by diminishing the supply of calcium‐permeable subunits for assembly into AMPAR. In contrast, recent findings indicating a role for AMPAR subunits GluA2, 3, and 4 in OPC survival (Kougioumtzidou et al., 2017) suggest that an increase in *Gria4* transcription, as observed in pOPC following excitotoxic treatment, may promote viability. Given this complexity, further studies are required to determine the potential of *Gria4* modulation to promote OPC viability under excitotoxicity‐inducing conditions such as hypoxic‐ischemia (Follett et al., 2000) and CNS inflammation (Groom et al., 2003; Kanwar et al., 2004; Pitt et al., 2000).

### 4.5 Involvement of NF‐Y in cell death

Both excitotoxic injury and pharmacological inhibition of NF‐Y reduced the function of this transcription factor and compromised viability in Oli‐neu cells, while siRNA‐mediated knockdown of *NF‐Yb* similarly reduced viability in cultures of pOPC. Disruption of NF‐Y function also induced apoptosis and regulated a core group of apoptotic genes that were similarly regulated by excitotoxicity. Together these findings suggest a link between NF‐Y, oligodendroglial survival, and the regulation of genes controlling apoptosis. In agreement with this, a number of reports provide evidence for the involvement of NF‐Yb in anti‐apoptotic actions. For example, NF‐Yb knockdown in a colorectal carcinoma cell line (HCT116) induces apoptosis through reductions in the anti‐apoptotic genes Bcl‐2 and BI‐1 (Benatti et al., 2008). ChIP assays and luciferase reporter experiments show that Bcl‐2 and BI‐1 are targets of NF‐Yb and that reduced NF‐Y function decreases transcription of these genes, indicating that NF‐Yb directs the activation of these anti‐apoptotic genes (Benatti et al., 2008). In the same study it was reported that reduction of NF‐Yb leads to increased acetylation of p53, a condition that promotes apoptosis *via* increased p53 binding to pro‐apoptotic targets Bax and Mdm2. Thus, NF‐Yb may regulate the apoptotic response of cells by controlling the balance between pro‐ and anti‐apoptotic pathways. In line with this idea, recent work in a human osteosarcoma cell line (U2OS) identified NF‐Yb as a direct target of pro‐apoptotic E2F1 (Jiang, Nevins, Shats, & Chi, 2015). Importantly, knockdown of *NF‐Yb* enhances E2F1‐mediated activation of pro‐apoptotic targets and increases apoptosis.

Increasing expression of NF‐Yb may not promote survival under all conditions of cellular stress. For example, the pro‐apoptotic FAS/APO1 gene is a direct NF‐Y target, whose expression in a breast cancer cell line (MCF7) is enhanced by both apoptotic DNA damage, and the forced expression of NF‐Y proteins (Morachis, Murawsky, & Emerson, 2010). Similarly, forced transduction of carcinoma cell line NT2/D1 with NF‐Ya proteins reduces cellular growth by inducing a decrease in their survival (Mojsin, Topalovic, Marjanovic Vicentic, & Stevanovic, 2015). The relationship between NF‐Y function and cell viability therefore appears to be complex, with the effects of loss and gain of NF‐Y function varying under different cellular and molecular contexts. Our experiments in pOPC revealed an increase in the transcription of *NF‐Yb* following excitotoxic injury, yet, at the same time knockdown of *NF‐Yb* decreased their viability, as did excitotoxic injury in Oli‐neu. Although appearing contradictory, these outcomes are consistent within the framework of NF‐Y function discussed above, where roles in the regulation of both pro‐ and anti‐apoptotic responses have been described (Gatta, Dolfini, & Mantovani, 2011). Importantly, the present data clearly show that dysregulation of NF‐Yb consistently associates with reduced pOPC viability; therefore the direct modulation of NF‐Y function is unlikely to provide a viable strategy for protecting OPC from excitotoxic injury.

Alterations in NF‐Y function per se are likely to compromise OPC survial *in vivo*, therefore we performed a network analysis on gene expression microarrays to examine the transcriptional events associated with NF‐Y in oligodendroglia. This analysis in Oli‐neu subjected to excitotoxic injury and pharmacological disruption of NF‐Y revealed that 43% of the genes commonly regulated in these models were associated with the regulation of apoptosis. On this basis these genes, and their downstream effectors, may provide promising targets for future work aiming to identify targets capable of protecting OPC from excitotoxic injury without compromising other essential NF‐Y functions.

### 4.6 Involvement of NF‐Y in CNS dysfunction

As discussed above our data suggest an important role for NF‐Y function in OPC survival. This conclusion is complemented by studies indicating NF‐Y deregulation as a contributor to neurodegenerative processes in several polyglutamine (polyQ) expansion diseases (Huang, Ling, Yang, Li, & Li, 2011; Katsuno et al., 2010; Yamanaka et al., 2008). In each case, disrupted NF‐Y activity is associated with the accumulation of mutated polyQ‐bearing proteins. Neurodegeneration then proceeds, in part, due to alterations in the transcription of NF‐Y targets required for neuronal survival. This is exemplified in spinal and bulbar muscular atrophy, a polyQ disorder involving the accumulation of mutated androgen receptor (AR) protein in brain stem and spinal cord motorneurons. In this condition transcription of transforming growth factor‐ß receptor type‐II (TGFßR‐II), a pro‐survival NF‐Y target, is impaired due to abnormal associations between NF‐Y and mutated AR that probably prevent NF‐Y from binding and activating TGFßR‐II (Katsuno et al., 2010). Similarly, mutant Huntington, the pathological protein underlying Huntington's Disease (HD), forms inappropriate interactions with NF‐Y leading to reduced transcription of heat shock protein 70 (HSP70), an NF‐Y target expected to aid in the clearance of mutated proteins. These links between NF‐Y dysfunction and CNS pathology and our present data suggesting anti‐ and pro‐survival roles in oligodendroglia, highlight NF‐Y dysfunction as an interesting target for evaluation in pathological conditions involving oligodendrocyte and myelin degeneration, including those mediated through excitotoxic injury (Groom et al., 2003; Kanwar et al., 2004; Pitt et al., 2000).

In conclusion, this work identifies NF‐Yb as a key controller of *Gria4* expression in OPC. AMPA‐type glutamate receptors exert a powerful influence on the development and survival of OPC (Fannon et al., 2015; Gallo et al., 1996; Kougioumtzidou et al., 2017; Yuan et al., 1998), and are themselves under strong developmental regulation (Hossain et al., 2014; Itoh et al., 2002), thus further work exploring the interplay between NF‐Y function and OPC differentiation represent a promising line of enquiry for studies aiming to uncover novel mechanisms regulating myelination. This work also highlights the NF‐Y system as an important regulator of OPC survival during excitotoxic injury. OPC are exquisitely vulnerable to hypoxic‐ischemic conditions (Back et al., 2002; Back et al., 2001; Back & Rosenberg, 2014). Therefore, we consider the NF‐Y network as an interesting focus for future work aiming to identify novel therapeutic targets for the protection of OPC during excitotoxic injury.

## 5 ORIGINAL DATA FILES

The transcriptomics data discussed in this publication including the complete single channel analysis are available at the following URL: http://epapers.bham.ac.uk/3088/).

## Supporting information

Additional Supporting Information may be found online in the supporting information tab for this article.

Supporting Information Figure S1

Supporting Information Figure S2

Supporting Information Figure S3

Supporting Information Figure S4

Supporting Information Table S1

Supporting Information Table S2

Supporting Information Table S3

Supporting Information Table S4‐8

Supplementary Tables and Figure Legends
