# Supplementary figures and images for: NF‐Y‐dependent regulation of glutamate receptor 4 expression and cell survival in cells of the oligodendrocyte lineage
Source: Glia. 2018 Apr 27;66(9):1896–914. doi: 10.1002/glia.23446 (PMC6220837; doi:10.1002/glia.23446)

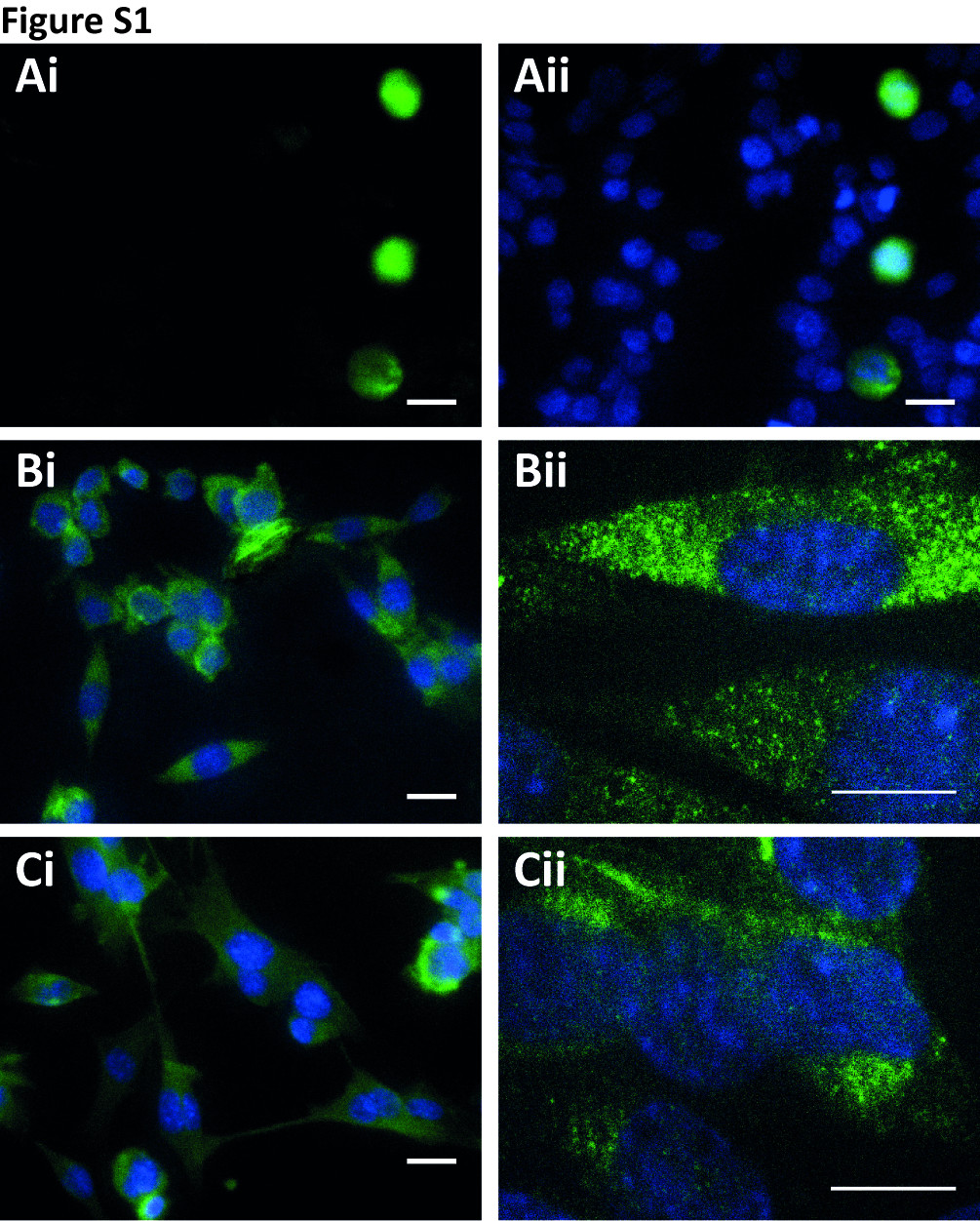

Supplement: Supplementary file 1 — Supporting Information Figure S1 [file GLIA-66-1896-s001.tif]

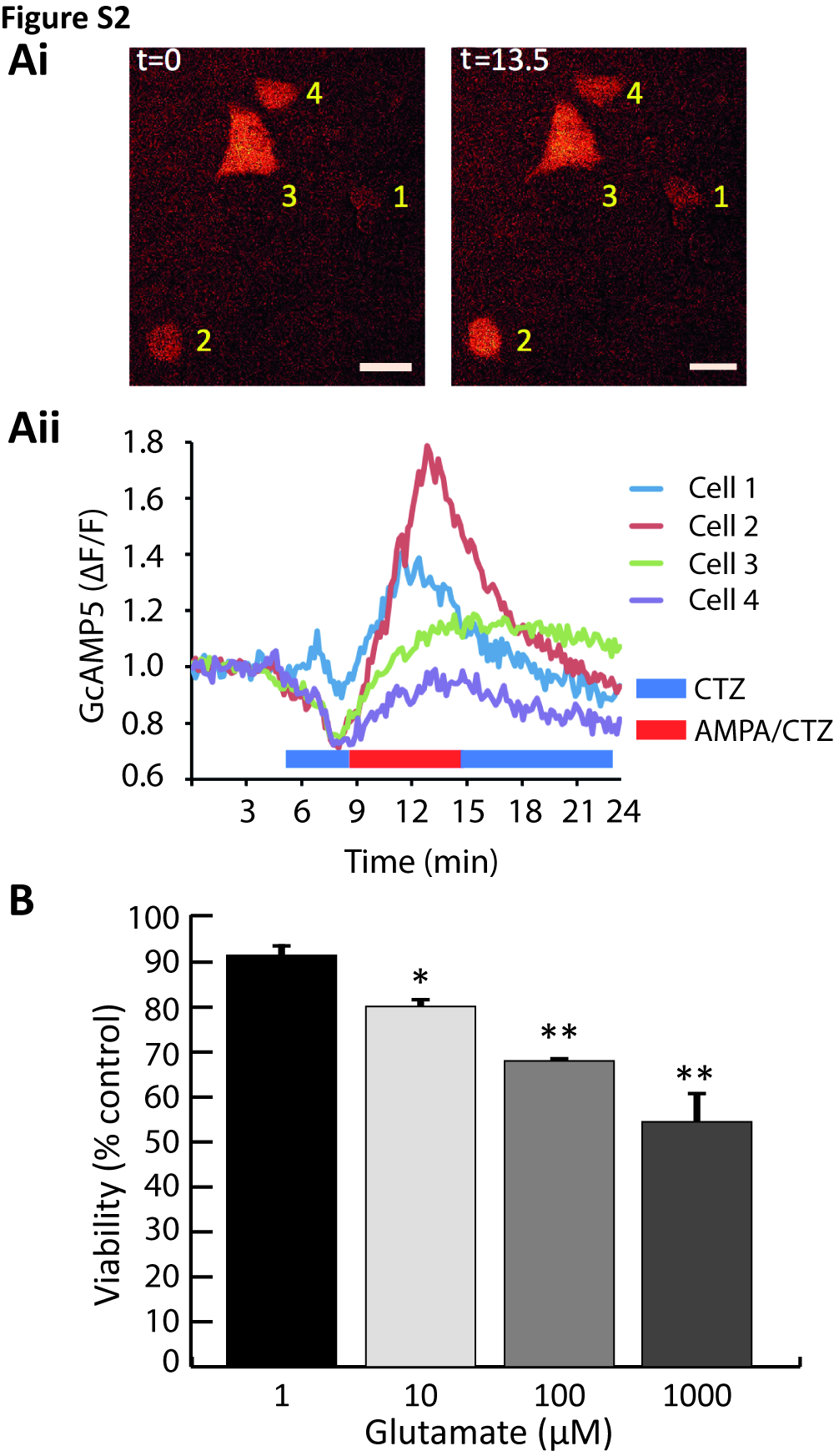

Supplement: Supplementary file 2 — Supporting Information Figure S2 [file GLIA-66-1896-s002.tif]

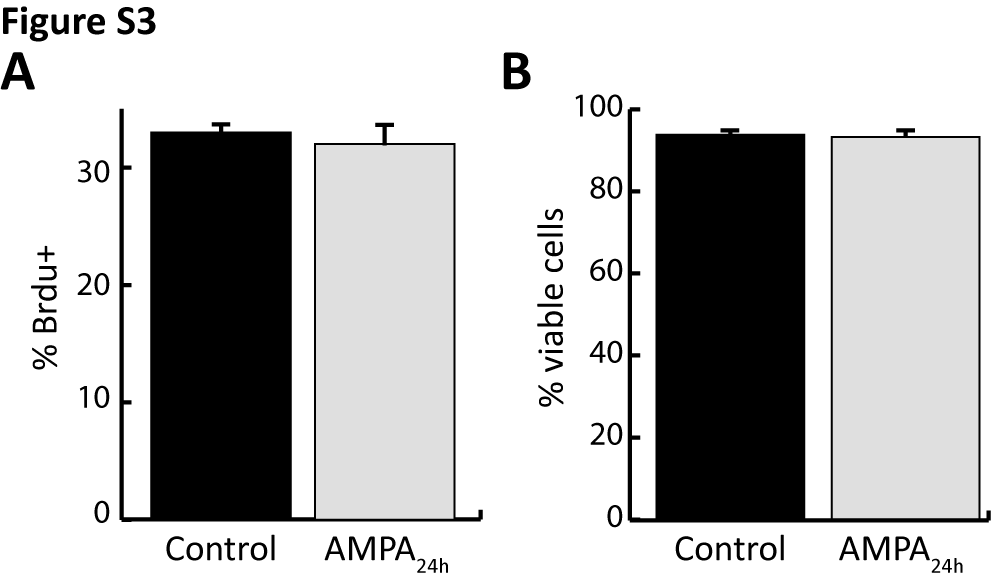

Supplement: Supplementary file 3 — Supporting Information Figure S3 [file GLIA-66-1896-s003.tif]

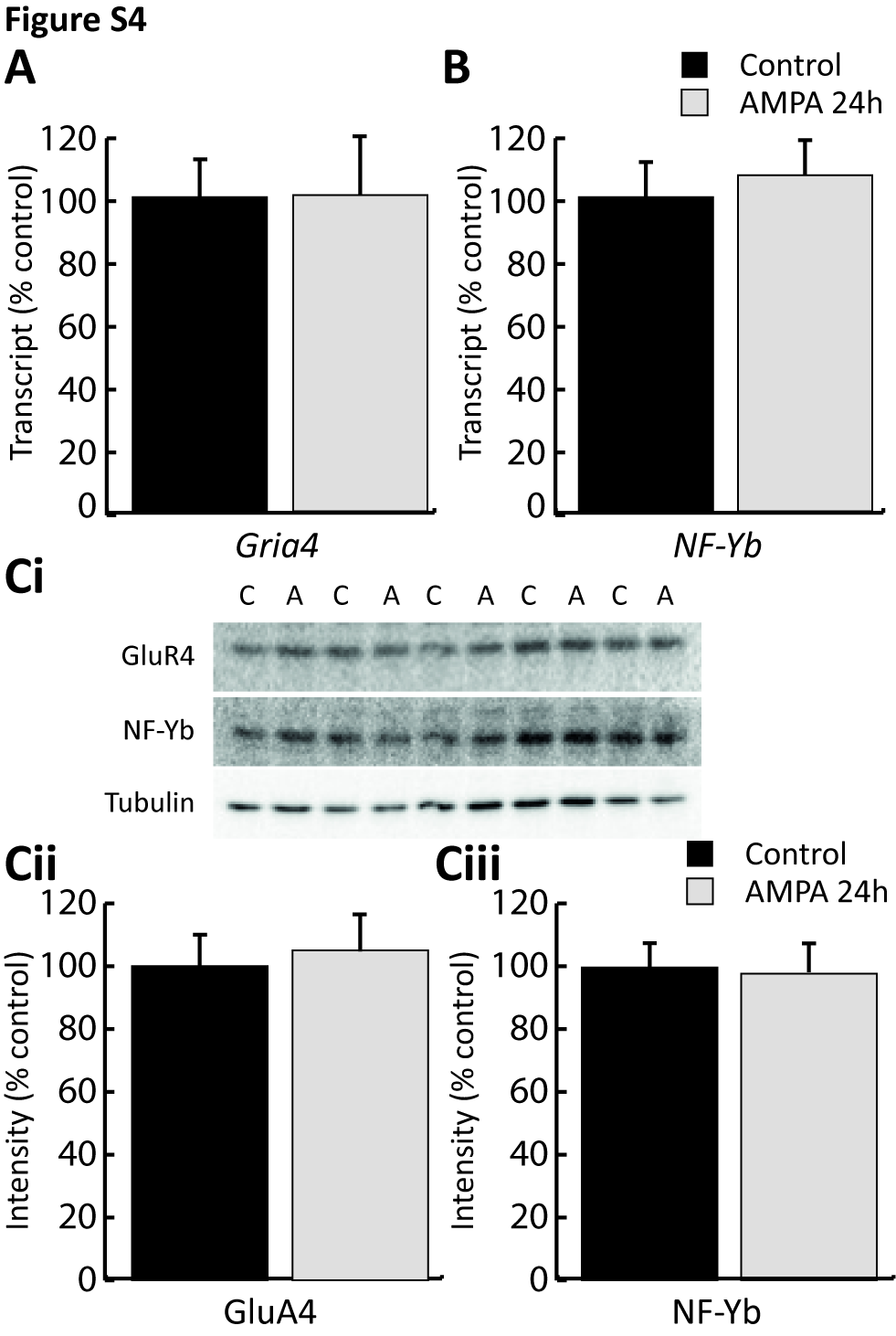

Supplement: Supplementary file 4 — Supporting Information Figure S4 [file GLIA-66-1896-s004.tif]
