# Supplementary material for: NF‐Y‐dependent regulation of glutamate receptor 4 expression and cell survival in cells of the oligodendrocyte lineage
Source: Glia. 2018 Apr 27;66(9):1896–914. doi: 10.1002/glia.23446 (PMC6220837; doi:10.1002/glia.23446)
Supplement: Supplementary file 5 — Supporting Information Table S1 [file GLIA-66-1896-s005.docx]

| **Supplementary Table 1. Antibodies** | | | | | | | |
| --- | --- | --- | --- | --- | --- | --- | --- |
| **Antibody** | **Host** | **Type** | **Supplier** | **Catalogue#** | **IF dilution** | **WB dilution** | |
| Anti-GluA1 | Rabbit | Polyclonal | Merck Millipore | AB1504 | 1/200 | --------- | |
| Anti-GluA2 | Mouse | Monoclonal | Merck Millipore | MAB397 | 1/200 | --------- | |
| Anti-GluA3 | Rabbit | Polyclonal | Alamone Laboratories | AGC101 | 1/200 | --------- | |
| Anti-GluA4 | Rabbit | Polyclonal | Merck Millipore | AB1508 | 1/200 | 1/2,000 | |
| Anti-Cleaved Caspase-3 | Rabbit | Polyclonal | Abcam | Asp175 | 1/400 | --------- | |
| Anti-NF-Yb | Rabbit | Polyclonal | Abcam | AB123384 | -------- | 1/500 | |
| Anti-α-Tubulin | Rabbit | Polyclonal | New England BioLabs | 2144S | -------- | 1/5,000 | |
| Anti-GAPDH | Rabbit | Polyclonal | Abcam | AB9485 | -------- | 1/5,000 | |
| Anti-BrdU | Rat | Polyclonal | Abcam | AB6326 | 1/200 | --------- | |
| Alexa fluor 594 ant-rabbit IgG | Goat | Polyclonal | Thermo Fisher | A11037 | 1/800 | --------- | |
| Alexa fluor 488 ant-mouse IgG | Goat | Polyclonal | Thermo Fisher | A11029 | 1/800 | --------- | |
| Alexa fluor 594 ant-mouse IgG | Goat | Polyclonal | Thermo Fisher | A11032 | 1/800 | --------- | |
| HRP conjugate anti-Rabbit IgG | Goat | Polyclonal | Promega Corporation | W4011 | -------- | 1/5,000 | |
| HRP = horseradish peroxidase. IF = immunofluorescence. WB = Western blot. | | | | | | |  |
