# Supplementary material for: NF‐Y‐dependent regulation of glutamate receptor 4 expression and cell survival in cells of the oligodendrocyte lineage
Source: Glia. 2018 Apr 27;66(9):1896–914. doi: 10.1002/glia.23446 (PMC6220837; doi:10.1002/glia.23446)
Supplement: Supplementary file 6 — Supporting Information Table S2 [file GLIA-66-1896-s006.docx]

| **Supplementary Table 2. Sequence information** | | |
| --- | --- | --- |
| **Gene expression and ChiP primers** | | |
| **Name of Gene** | **Forward primer (5' to 3')** | **Reverse primer (3' to 5')** |
| *Gria4* | GTTTTCTGGATTTTGGGGACTCG | AAGAGACCACCTATTTGAACGC |
| *Nfyb* | GCCTCCCAGCTAGGGATTTC | TTCCTGTTTGAGGTATGGCAT TT |
| *Nfyb* CCAAT site 1 | CATTGGTCTTGAGCCTTTCA | CATTGGTCTTGAGCCTTTCA |
| *Nfyb* CCAAT site 2 | TCATCCCAAGATTTTCCTTTGGA | CCAAATAGCCCTAACACTTTCTC |
| *Nfyb* CCAAT site 3 | CTGCCAATTTGCTAGGCATT | CCCACATTGCTCATATTTCCA |
| *ACTB* | ACCTTCTACAATGAGCTGCG | CTGGATGGCTACGTACATGG |
| All sequences are against mouse genes. | | |
