# Supplementary material for: NF‐Y‐dependent regulation of glutamate receptor 4 expression and cell survival in cells of the oligodendrocyte lineage
Source: Glia. 2018 Apr 27;66(9):1896–914. doi: 10.1002/glia.23446 (PMC6220837; doi:10.1002/glia.23446)
Supplement: Supplementary file 7 — Supporting Information Table S3 [file GLIA-66-1896-s007.docx]

| **Supplementary Table 3. *Gria4* NF-Yb binding sites** | | |
| --- | --- | --- |
| ***Nfyb* binding site** | **Wild type site** | **Mutated site** |
| *Nfyb* site 1 | CAAGA**CCAAT**GTTGTT | CAAGAtcgatGTTGT |
| *Nfyb* site 2 | GTGCA**CCAAT**GCTTA | GTGCAgatcaGCTTA |
| *Nfyb* site 3 | ACCTG**CCAAT**TTGCT | ACCTGtagcaTTGCT |
| Wildtype sites: Bold underlined nucleotides. Mutated sites: Lower case underlined nucleotides. | | |
