# Supplementary material for: NF‐Y‐dependent regulation of glutamate receptor 4 expression and cell survival in cells of the oligodendrocyte lineage
Source: Glia. 2018 Apr 27;66(9):1896–914. doi: 10.1002/glia.23446 (PMC6220837; doi:10.1002/glia.23446)
Supplement: Supplementary file 9 — Supplementary Tables and Figure Legends [file GLIA-66-1896-s009.docx]

**SUPPLEMENTARY TABLES AND FIGURE LEGENDS**

**Supplementary Table S1.** List of antibodies used in the study.

**Supplementary Table S2.** List of primers used for qPCR and ChiP studies.

**Supplementary Table S3.** NF-Yb binding sites in mouse *Gria4*.

**Supplementary Table S4.** Transcriptomic analysis from Agilent two-colour microarrays for AMPA/CTZ vs CTZ (*P*<0.01). Output from separate channel analysis.

**Supplementary Table S5.** Transcriptomic analysis from Agilent two-colour microarrays for Garcinol vs DMSO vehicle control (*P*<0.01). Output from separate channel analysis.

**Supplementary Table S6.** Interactome model for AMPA/CTZ vs CTZ, output from Moduland algorithm.

**Supplementary Table S7.** Interactome model for Garcinol vs DMSO vehicle control, output from Moduland algorithm.

**Supplementary Table S8.** Overlap of genes between network model core hierarchy (unique sum of the central ten genes of each of the top 30 modules).

**Supplementary Figure 1.**  Expression of AMPAR subunits GluA1-3 in Oli-neu cells. **A**. GluA1 expression in Oli-neu cells. **Ai**. Single-channel immunofluorescent image of GluA1 (green) suggested nuclear localization. **Aii**. Merged image for GluA1 (green) and DAPI (blue) confirmed nuclear localization, and indicated that GluA1 is expressed in a minority of Oli-neu cells. **B-C**. Immunofluorescent labeling for GluA2 (**B**) and GluA3 (**C**). **Bi, Ci.** Merged images for GluA2 and GluA3 respectively (green), and DAPI (blue) at 20x. **Bii, Cii**. 100x images reveal punctate nature of GluA2 and GluA3 expression in Oli-neu cells. Scale bars in Ai-ii, Bi, Ci, =20µm. Scale bars in Bii, Cii, =10µm.

**Supplementary Figure 2**. Oli-neu cells expressed functional glutamate receptors. **A**. Glutamate exposure caused a concentration-dependent reduction in Oli-neu cell viability as measured by the Trypan blue assay. Cell viability (% of viable cells normalized to the non-glutamate treated control) was significantly reduced after 5 hour exposures to 10µM (80.1 ± 1.5%), 100 µM (68.1 ± 0.4%) and 1000µM (54.5 ± 6.2%) glutamate. **B**. Activation of AMPAR evoked elevations in intracellular Ca^2+^ levels in Oli-neu cells. **Bi**. GcAMP5 signals from 4 Oli-neu cells recorded before application of AMPA/CTZ (t=0) and during the peak of the response (t=13.5). Scale bar = 20µm. **Bii**. Quantification of GcAMP5 signals (ΔF/F) recorded from the cells depicted in Bi. Unambiguous elevations in intracellular Ca^2+^ are apparent in 50% of the cells depicted in Bi. * and ** Significance *P*< 0.05 and *P* < 0.01 respectively. Data expressed as means ± SEM.

**Supplementary Figure 3**. Prolonged activation of AMPAR in the absence of CTZ did not affect Oli-neu proliferation or viability. **A**. Proliferation measured by BrdU incorporation did not differ between control and AMPA_24h_ treated cells (Control 33.2 ± 0.95%, AMPA_24h_ 32.2 ± 1.76%). **B**. Cell viability measured by the Trypan blue assay was comparable in control and AMPA_24h_ treated cultures (Control 93.8 ± 0.55%, AMPA_24h_ 93.3 ± 0.17%). Data expressed as means ± SEM.

**Supplementary Figure 4**. Prolonged activation of AMPAR in the absence of CTZ did not alter the expression of GluA4 or NF-Yb. **A, B**. Transcript levels for *Gria4* (**A**) and *NF-Yb* (**B**) were unaffected by AMPA_24h_ treatment (GluA4: Control 1.00 ± 0.12, AMPA_24h_ 1.01 ± 0.19; NF-Yb: Control 1.00 ± 0.11, AMPA_24h_ 1.07 ± 0.11). **C.** Western blot analysis from Oli-neu cells treated with AMPA_24h_**.** **Ci.** Representative immunoblots for GluA4 (top), NF-Yb (middle) and α-tubulin (bottom) from 4 independent experiments. **Cii, Ciii**. Densitometric analysis of immunoblots shown in Ci (normalized against α-tubulin) indicated reduced levels of GluA4 (**Cii**) and NF-Yb (**Ciii**) protein after exposure to AMPA_24h_ (GluA4: Control 1.00 ± 0.05, AMPA_24h_ 0.76 ± 0.04; NF-Yb: Control 1.00 ± 0.12, AMPA_24h_ 0.69 ± 0.08). Data expressed as means ± SEM.
